# Online Monitoring and Damage-Oriented Health Management of Multi-Disc Clutch Considering Digital Twin: A Review

**DOI:** 10.3390/ma19214579

**Published:** 2026-10-29

**Authors:** Biao Ma, Dingge Zhang, Liang Yu, Yuqing Feng, Xin Ao, Yiyang Zheng

**Affiliations:** 1 School of Mechanical Engineering, Beijing Institute of Technology, Beijing 100081, China; mabiao@bit.edu.cn (B.M.); 3120235496@bit.edu.cn (D.Z.); 3220240407@bit.edu.cn (X.A.); 3220250507@bit.edu.cn (Y.Z.); 2 State Key Laboratory of Mechanical Transmission for Advanced Equipment, Chongqing University, Chongqing 400044, China; 3 Weichai Power Co., Ltd., Weifang 261001, China; fengyuqing@weichai.com

**Keywords:** multi-disc clutch, wear, damage evolution, digital twin, failure analysis

## Abstract

**Highlights:**

**What are the main findings?**

- Recent advances in multi-disc clutch damage mechanisms are reviewed.
- Damage indicators linking degradation and failure are summarized.
- A conceptual self-evolving DT framework for clutch health management is synthesized.

**What are the implications of the main findings?**

- Damage evolution provides a basis for condition assessment and prognosis.
- Digital twin technology enables damage-aware monitoring throughout service life.
- The framework guides future DT-based clutch health management research.

**Abstract:**

The increasing demands for safety, comfort, and reliability of automotive transmission systems have highlighted the need for effective in-service health management of multi-disc clutches. Under severe thermo-mechanical conditions, friction components experience progressive degradation and irreversible damage, including wear, thermal deterioration, structural instability, and fatigue cracking, which may ultimately lead to failure. This paper systematically reviews recent advances in friction-wear behavior, thermal load, dynamic response, and damage-related failure mechanisms from a damage-oriented perspective, with particular attention to damage classification, evolution, and quantitative indicators. The development and representative applications of digital twin (DT) technology are subsequently reviewed. On this basis, a conceptual self-evolving digital twin (SEDT) framework comprising a physical entity, virtual model, data and software system, and knowledge base is synthesized for multi-disc clutches. The framework integrates damage-aware sensing and modeling with multi-source data fusion, diagnosis, prognosis, and maintenance decision making, and its prospective application to wear prediction under varying loads is illustrated. Finally, current limitations, implementation challenges, and future research directions for SEDT-based clutch health management are discussed.

## 1. Introduction

The automotive industry is a major contributor to the global economy, and automobiles remain an essential mode of transportation. The rapid development of conventional, electrified, and intelligent vehicles is imposing increasingly stringent requirements on transmission systems in terms of response speed, ride comfort, reliability, and durability [1,2,3]. As a key component of automotive transmission systems, the multi-disc clutch is widely used because of its stable operation, high torque-transmission capacity, and suitability for automatic control [2,4]. During service, multi-disc clutches are subjected to severe thermo-mechanical operating conditions involving high temperatures and contact pressures [5,6]. Repeated engagement and prolonged operation progressively degrade the performance of the friction components [7,8]. Such degradation can shorten clutch service life, compromise driving safety, and limit further improvements in transmission reliability [9]. Therefore, online clutch monitoring is particularly necessary to quantify its performance state and remaining useful life during service [10,11].

Effective health management is also essential for extending clutch service life and maintaining operational reliability [12,13]. Accordingly, equipment health management is shifting from reactive fault diagnosis toward proactive prognosis and predictive maintenance [14,15]. However, several limitations remain in existing clutch health-management approaches. Multi-disc clutch operation involves strongly coupled mechanical, thermal, hydraulic, and tribological processes, whereas many existing approaches focus primarily on system-level performance indicators without explicitly characterizing the underlying damage evolution [16]. In addition, data-driven health-management methods often rely heavily on historical failure data, which limits their applicability when representative failure samples are unavailable [17]. Many existing approaches also lack the capability to dynamically update health assessments and maintenance decisions in response to changing operating conditions [18]. These limitations motivate the development of a more adaptive health-management approach capable of integrating multi-physics knowledge, real-time monitoring data, and damage evolution.

**Figure 1:**
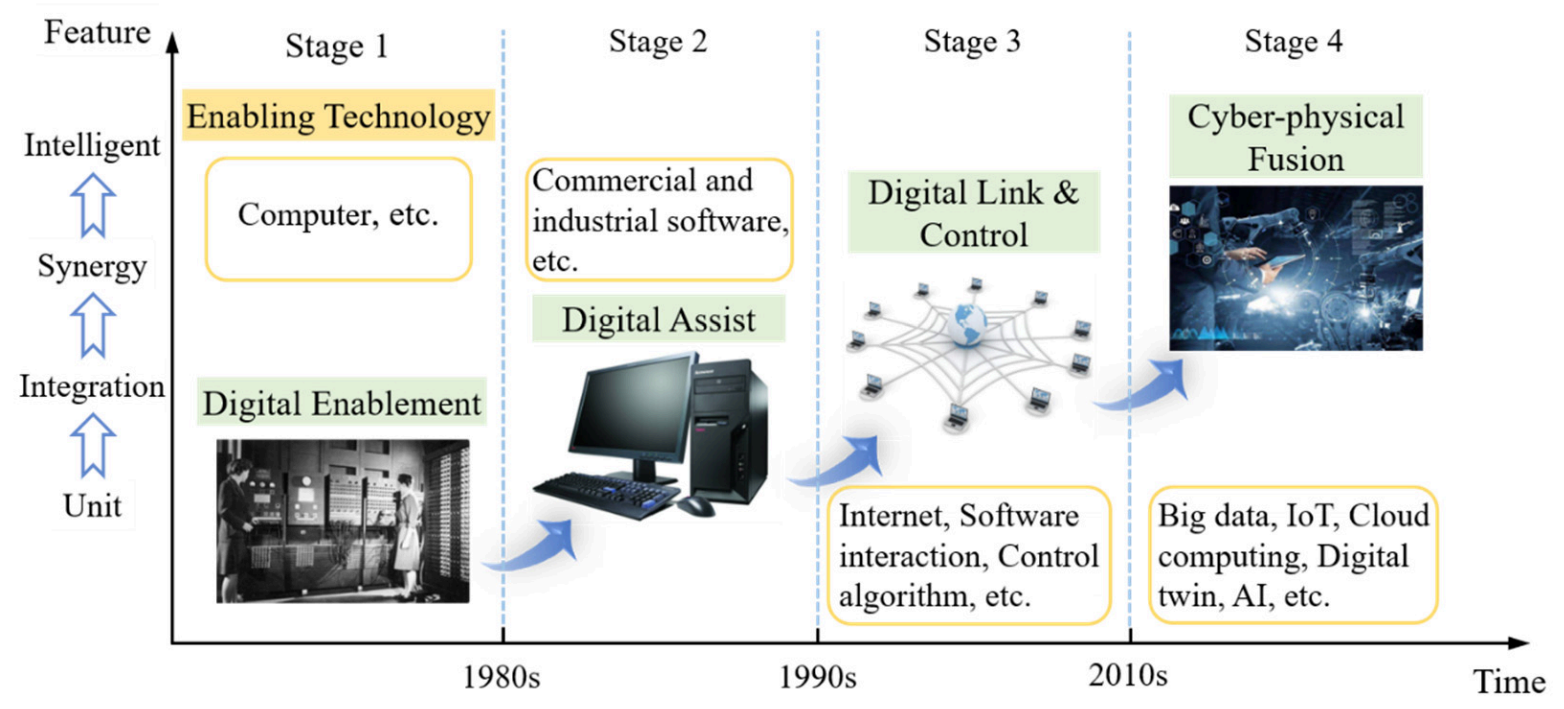
Evolution of digitalization and digital twin technology (adapted and reorganized from Qi et al. [19]).

As shown in Figure 1, adapted and reorganized from Qi et al. [19], the digitalization revolution can be broadly characterized by increasing levels of physical–digital integration, progressing from digital enablement and assistance to digital connectivity, control, and ultimately cyber–physical fusion [19,20,21]. Technologies such as big data analytics, the Internet of Things (IoT), cloud computing, artificial intelligence, and advanced communication networks have accelerated this transition by enabling continuous interaction between physical assets and their digital representations [19,22,23,24]. Within this context, digital twin (DT) technology has emerged as a promising approach for cyber–physical integration [25]. DT provides a potential means of integrating physical systems, virtual models, and operational data throughout the system lifecycle [26,27].

The concept of digital twin (DT) was initially developed in the context of product lifecycle management and has subsequently evolved into an important paradigm for cyber-physical integration. Grieves introduced the early concept of a digital representation of physical products, emphasizing the interaction among physical entities, virtual models, and data connections [28]. Subsequently, NASA and the U.S. Air Force Research Laboratory further advanced DT applications in aerospace engineering by integrating high-fidelity simulations, sensor information, and historical operational data for structural health monitoring and life prediction [29,30]. With the advancement of sensing technologies, data analytics, and artificial intelligence, DT has gradually expanded from aerospace systems to industrial equipment, manufacturing, and prognostics and health management (PHM) [31]. These developments demonstrate the potential of DT to establish dynamic links between physical degradation mechanisms and operational states, thereby providing new opportunities for damage-aware monitoring and predictive maintenance of complex mechanical systems.

DT acts as an evolving digital replica of a physical entity, which provides an evolving digital representation from design to the end of the service cycle [32]. By integrating operational data with virtual models, a DT can reproduce system behavior and enable real-time interaction, model updating, and optimization between the physical and virtual spaces [33]. As a consequence, the mechanism and application mode of DT have been extensively explored in various fields, including equipment development, damage prognosis, and health management [34,35,36]. However, systematic DT frameworks explicitly oriented toward damage evolution and health management of multi-disc clutches remain limited. An appropriate DT architecture is therefore required to effectively integrate the physical system, virtual models, operational data, and health-management functions [37]. To address this gap, this review synthesizes a conceptual self-evolving digital twin (SEDT) framework for damage-oriented health management of multi-disc clutches. The framework is intended to establish a structured link among clutch damage mechanisms, online monitoring, prognosis, and maintenance decision making, while providing a reference for future DT implementation in related friction and transmission systems. The remainder of this review is organized as follows:

First, recent advances in multi-disc clutch research are reviewed from the perspectives of friction-wear behavior, thermal load, dynamic response, and damage-related failure mechanisms, and the corresponding research limitations are identified [38,39]. The origin, development, and representative applications of DT technology are then summarized, followed by the synthesis of a conceptual SEDT architecture comprising a physical entity, virtual model, data and software system, and knowledge base. Its prospective implementation for damage-oriented monitoring and health management of multi-disc clutches is subsequently discussed. Finally, the opportunities, challenges, limitations, and future research directions associated with SEDT-based clutch health management are discussed.

In this review, performance degradation is distinguished from material damage and ultimate failure. Degradation describes the gradual deterioration of performance-related properties during service, whereas damage refers to irreversible material or structural changes induced by frictional, thermal, or mechanical loading, such as wear, thermal deterioration, and structural instability. Failure corresponds to the final state in which accumulated damage results in the loss of required functional or load-carrying capability. Based on this distinction, the review emphasizes damage evolution as the key link between degradation and failure, particularly within DT–enabled monitoring and health management frameworks.

## 2. Literature Review of Multi-Disc Clutches

### 2.1. Structure, Operating Principle, and Damage Classification of Multi-Disc Clutches

**Figure 2:**
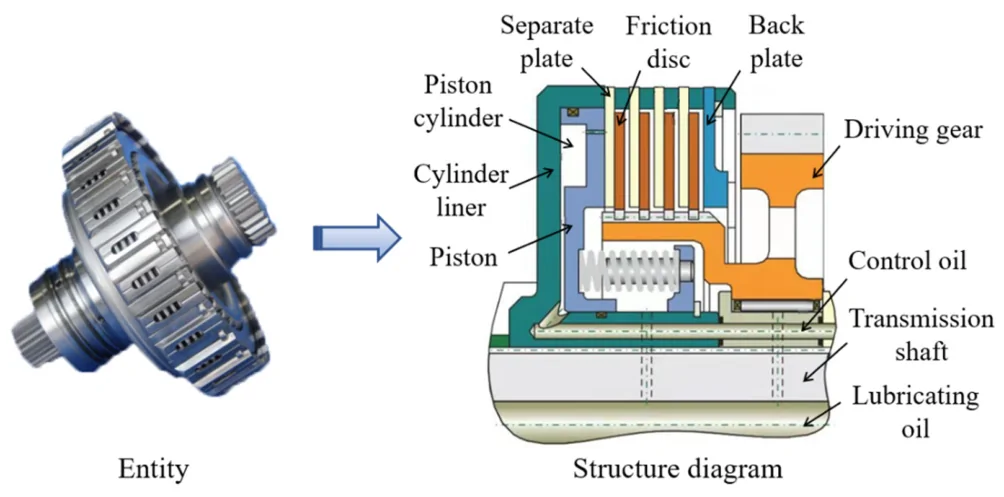
Illustration of a multi-disc clutch (structure diagram adapted from Ref. [40]).

As shown in Figure 2, a multi-disc clutch mainly consists of a piston, friction discs, separator plates, a back plate, a retaining component, and the associated hydraulic and lubrication systems. The friction discs and separator plates are alternately arranged to form multiple friction pairs. The friction discs are connected to one rotating member through splines, whereas the separator plates are connected to the other, allowing torque to be transferred between the driving and driven components during engagement.

During clutch engagement, hydraulic pressure drives the piston to compress the friction pack, progressively reducing the clearance between adjacent friction components. The resulting normal force promotes frictional contact between the friction discs and separator plates, through which torque is transferred from the driving side to the driven side until their relative sliding speed approaches zero. During this process, the friction interface undergoes transient changes in lubrication, contact pressure, sliding speed, frictional heat generation, and temperature. During disengagement, the applied pressure is released and the friction pairs are separated, interrupting torque transmission.

As the primary torque-transmitting elements, the friction components play a critical role in determining clutch engagement performance and service reliability. Accordingly, extensive theoretical and experimental studies have been conducted on their operating behavior, damage mechanisms, condition monitoring, and health management. The existing research is therefore reviewed from four interconnected perspectives: friction-wear behavior, thermal load, dynamic response, and failure mechanisms, as summarized in Table 1.

For damage-oriented health management, it is necessary to distinguish performance degradation, material damage, and functional failure. Performance degradation refers to a sustained deterioration in clutch functional performance, which may be reflected in changes in the coefficient of friction (COF), torque-transfer characteristics, or engagement time. However, these changes do not necessarily indicate irreversible damage, as clutch performance is also affected by operating pressure, sliding speed, temperature, and lubrication conditions. In contrast, damage refers to irreversible material or structural changes, such as wear-induced material loss, permanent thermal deterioration, plastic deformation, and fatigue cracking. Failure occurs when the clutch can no longer satisfy specified functional or safety requirements, such as the required torque-transfer capability or acceptable engagement performance. Therefore, performance degradation indicators, physical damage variables, and functional failure criteria represent related but distinct aspects of clutch health.

In engineering monitoring, performance degradation can be quantitatively assessed through changes in COF, engagement time, friction torque, and thermal response under comparable operating conditions [10,11]. However, physical damage is often difficult to measure directly during clutch operation. Its assessment therefore requires the integration of damage-sensitive monitoring information with appropriate physical or data-driven models. Wear-related damage can be represented by wear depth, wear volume, or material loss [41,42,43], while thermal damage assessment requires consideration of temperature history and irreversible material changes. Structural damage can be characterized by permanent deformation or buckling amplitude, whereas fatigue damage can be described by crack dimensions and propagation behavior. Importantly, transient temperature increases, torque fluctuations, or vibration responses alone do not necessarily constitute evidence of irreversible damage. Such measurements should be interpreted together with mechanism-based models and, where available, experimental observations or inspection results.

Accordingly, engineering condition assessment can be organized into three interconnected steps. First, measurable performance indicators are evaluated against reference responses under corresponding operating conditions to identify sustained performance deterioration. Second, damage-related state variables are estimated using appropriate models and complementary monitoring information, with their reliability assessed through experimental calibration or validation. Third, the estimated performance and damage states are compared with component-specific functional or safety limits to assess failure risk. This distinction is particularly important because irreversible damage may accumulate before significant performance degradation becomes observable, while severe thermo-mechanical loading may also trigger rapid instability within a limited number of engagements. Consequently, the transition among degradation, damage, and failure should be evaluated in relation to the specific damage mechanism, operating conditions, and applicable assessment thresholds.

Existing studies characterize clutch damage using different criteria. Gong et al. classified the wear behavior of copper-based friction materials into ultra-mild, mild, and severe regimes based on measured wear rates [44], whereas Li et al. investigated two-stage wear evolution in paper-based friction materials under coupled thermal and mechanical effects [41]. These studies primarily characterize wear severity or progression under specific material and operating conditions. In contrast, Zagrodzki and Truncone investigated thermoelastic instability (TEI) and hot-spot formation during transient clutch engagement [45], while Xiong et al. and Audebert et al. examined thermally induced buckling of friction components [46,47]. These studies focus on individual damage or instability mechanisms rather than a unified classification. Based on this literature, Table 2 provides a complementary classification according to the dominant evolution time scale, distinguishing progressive damage (wear, material deterioration, and fatigue accumulation) from rapid-onset phenomena (TEI-induced hot spotting and buckling). This classification is not universally established or mutually exclusive, but provides a common basis for comparing cumulative damage and transient instability. Progressive deterioration may increase susceptibility to rapid instability, while severe transient events may accelerate subsequent irreversible damage.

Although Table 2 summarizes representative damage indicators, their critical thresholds depend on friction materials, component geometry, lubrication conditions, and operating loads. Previous studies have established material- or configuration-specific boundaries through wear tests, stability analysis, and clutch experiments [44,45,46,48,49]. For a given clutch system, threshold determination should therefore involve: (1) selecting damage indicators and corresponding functional or safety criteria; (2) identifying critical conditions through experiments or validated physical models; and (3) calibrating the resulting thresholds against observed damage or failure states. Representative findings from the literature are summarized in Table 3. Importantly, the distinction between progressive and rapid-onset damage describes the dominant evolution time scale rather than a universal numerical transition boundary.

As an illustrative engineering scenario, consider a wet multi-disc clutch with copper-based friction material operating under the synthetic loading conditions described in Section 5.5. Suppose that its reference transmitted torque is 800 N·m under a specified operating condition, while the minimum required torque is 600 N·m. A sustained torque reduction exceeding 10% from the reference value (i.e., below 720 N·m) may be defined as a performance-degradation alert. Separately, a cumulative friction-lining wear depth of 20 μm may be adopted as an illustrative damage-severity alert, provided that the wear state is independently measured or appropriately calibrated. Using the synthetic wear observations in Section 5.5 (4, 13, and 29 μm), the wear alert would be exceeded after the third operating block. However, this threshold crossing alone does not indicate functional failure, which requires verification that the transmitted torque falls below 600 N·m or that another specified functional criterion is violated. These numerical criteria are hypothetical examples rather than experimentally established limits and must be calibrated for the specific clutch configuration and operating conditions.

Threshold uncertainty should also be considered in health assessment and maintenance planning. Variations in material properties, operating conditions, measurement accuracy, and model parameters may lead to uncertain estimates of damage onset or failure limits [43]. Such uncertainty can be evaluated through sensitivity analysis, statistical confidence intervals, or probabilistic modeling [43]. When combined with the estimated damage state, threshold uncertainty can be propagated into failure probability and remaining useful life predictions, allowing maintenance decisions to account for the associated risk rather than relying on a single deterministic limit.

Progressive damage and rapid-onset instability may interact through coupled frictional, thermal, and mechanical processes. Qiao et al. demonstrated that wear can either promote or suppress TEI, depending on friction-pair thickness and wear rate [51]. Conversely, TEI-induced hot spots may accelerate local material deterioration and frictional wear [41,45]. Thermal buckling can redistribute contact pressure, increasing local temperature and torque fluctuations [46,52]. Furthermore, studies on railway brake discs have shown that repeated hot spotting can promote thermal fatigue cracking [53,54,55], although this relationship requires further validation for wet multi-disc clutches. The representative interaction pathways and their supporting evidence are summarized in Table 4.

### 2.2. Friction-Wear

**Figure 3:**
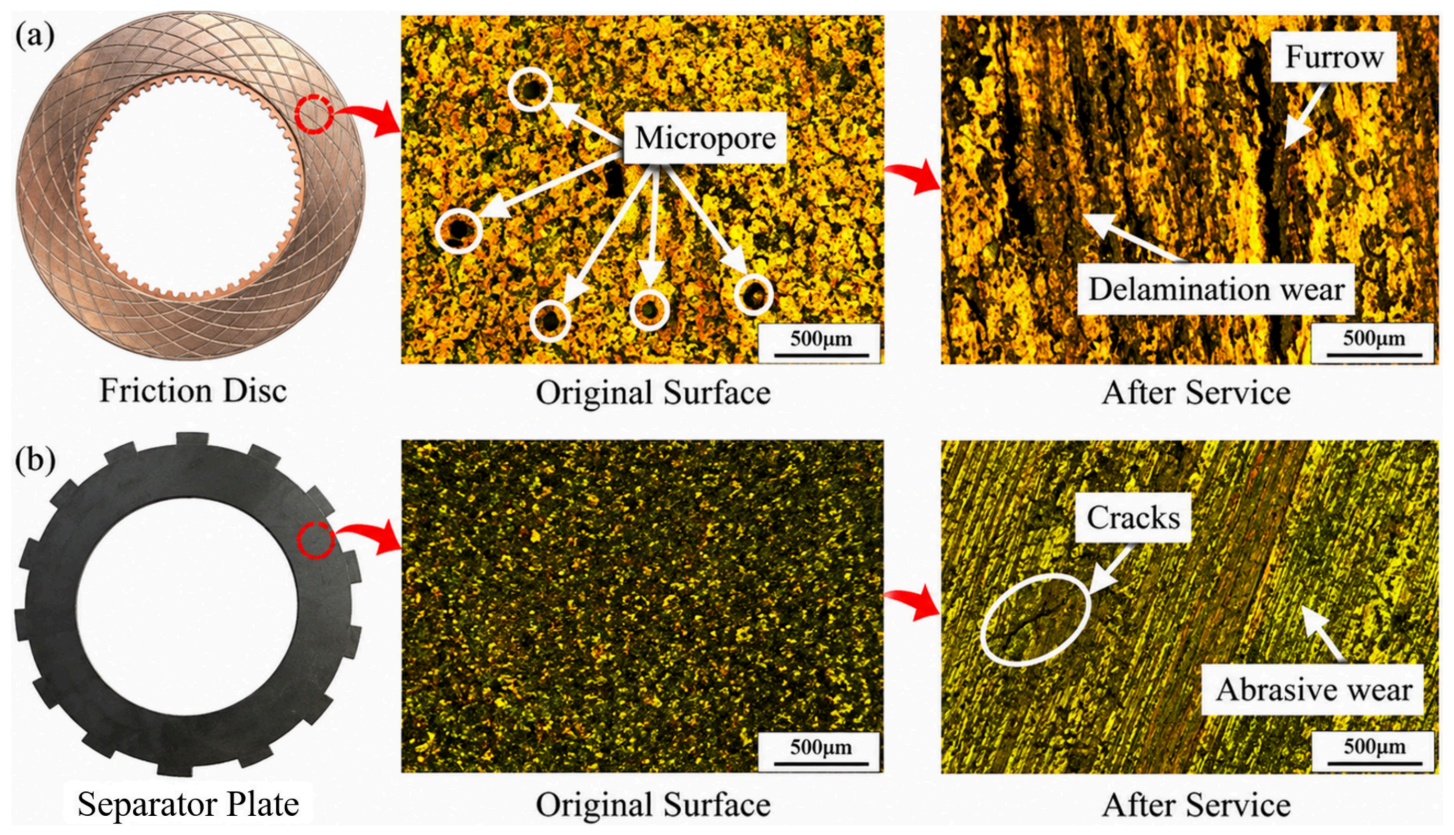
Representative wear characteristics and surface morphologies of the friction discs and separator plates: (**a**) friction disc and (**b**) separator plate [57].

Progressive material loss from friction surfaces leads to wear-induced damage and subsequent performance degradation [56]. Existing studies have investigated clutch wear from complementary perspectives, including wear-based remaining useful life prediction [56], thermohydrodynamic wear modeling [41], and model validation with uncertainty analysis [43]. These approaches provide different levels of physical detail but require material- and condition-specific calibration. As shown in Figure 3, representative manifestations of friction-induced damage include furrows, cracks, delamination, and abrasive wear [57].

#### 2.2.1. Material

Separator plates and friction-disc cores are commonly manufactured from 65Mn steel, whereas friction linings are typically composed of copper-based friction material (CFM) or paper-based friction material (PFM). Li et al. investigated the wear behavior of paper-based friction materials in wet clutches by considering both thermal degradation and mechanical effects. Their thermohydrodynamic-based wear prediction model successfully captured the experimentally observed two-stage wear behavior, highlighting the coupled influence of temperature history and contact stress on friction lining durability [41]. Carbon-based and ceramic friction materials have also attracted increasing attention for specialized applications [58,59,60,61]. Characterizing material condition provides a direct means of assessing wear severity and damage evolution. Generally, a wear map can be obtained for a specific material under specified working conditions to depict dominant wear processes and their severity. On this basis, Gong et al. classified the wear map of CFM from measured wear rates into ultra-mild, mild and severe wear states, and then discussed the dominant wear mechanisms [44]. Yu et al. conducted UMT-5 tests to compare the wear behavior of CFM and PFM over wide ranges of applied pressure, sliding speed, and ambient temperature, providing experimental support for condition assessment [62]. Additives can also significantly influence the friction and wear behavior of friction materials [63,64]. Lu et al. reported that an organosilicon content of approximately 7.5% provided the most stable COF for the investigated PFM [63].

#### 2.2.2. COF

The coefficient of friction (COF) is an important indicator of clutch friction performance, but its evolution is influenced by operating conditions and material changes. Jung et al. investigated friction characteristics during repeated clutch engagements [65], while Wu et al. investigated the effects of operating parameters on wet-clutch friction behavior and quantified their influences and interactions [66,67]. Farfan-Cabrera et al. further examined the influences of sliding distance and lubricant oxidation [68]. More recently, Strobl et al. demonstrated the importance of characterizing friction behavior across different sliding regimes and highlighted the measurement uncertainty associated with clutch testing [69].

For reliable condition monitoring, COF repeatability should be evaluated under comparable pressure, sliding speed, temperature, and running-in conditions, consistent with standardized testing procedures [70]. Repeated measurements can be characterized using statistical dispersion, while uncertainties associated with torque measurement, effective normal force, and instrument calibration should be assessed separately [71]. Importantly, measurement variability must be distinguished from actual COF changes caused by wear, temperature, or lubricant aging. Within a digital twin, these uncertainties should be considered during data fusion and model updating to avoid interpreting measurement fluctuations as physical degradation. Consequently, uncertainty-aware COF monitoring is essential for reliable clutch health assessment.

#### 2.2.3. Morphology

**Figure 4:**
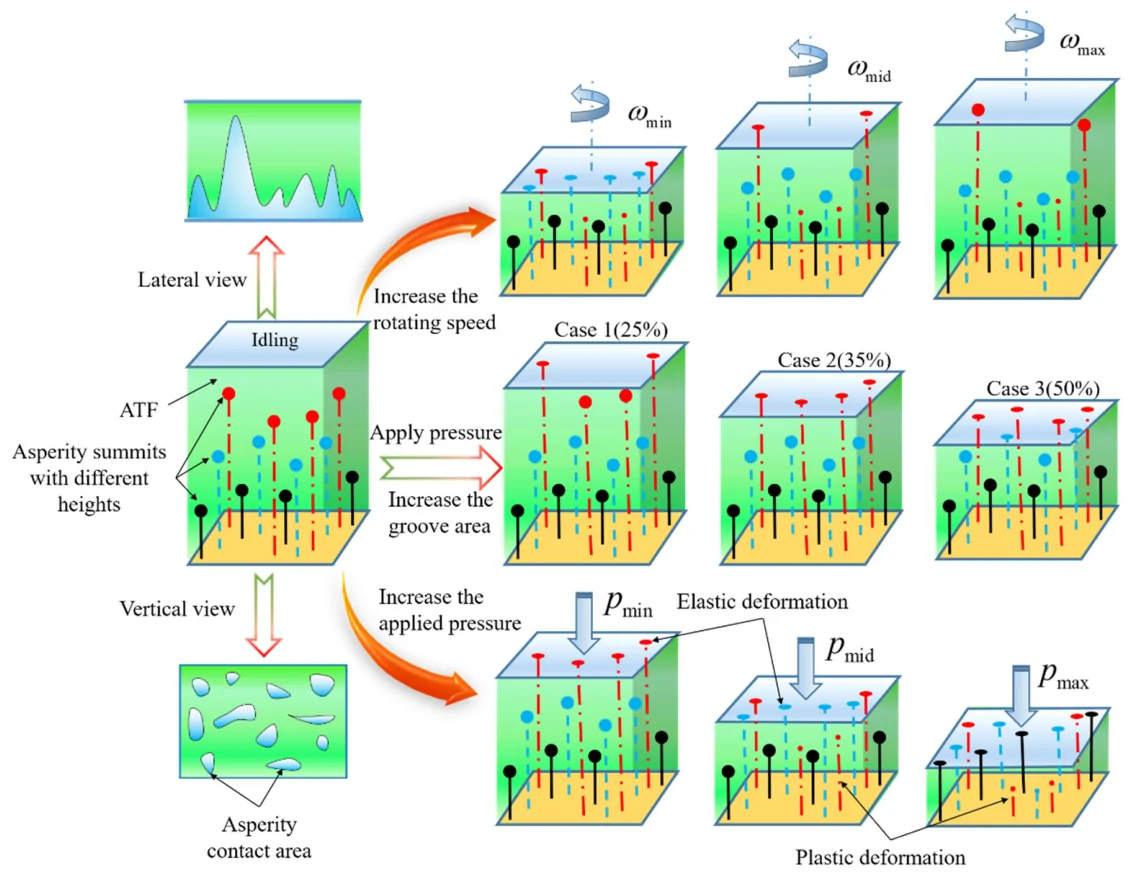
Micro-contact diagram of a friction pair [57].

Friction and wear continuously modify the surface topography of friction components, while the evolving topography in turn affects their subsequent tribological behavior [57], as shown in Figure 4. Li et al. further investigated the influence of groove patterns on friction lining durability through a thermo-hydrodynamic wear model, revealing the relationship between groove configuration, thermal response, and wear evolution [72]. To identify surface parameters capable of characterizing wear evolution, Nyman et al. examined the Birmingham parameters, namely the surface skewness (S_sk_), average slope (S_dq_), arithmetic mean summit curvature (S_sc_) and surface bearing index (S_bi_) [73]. Their results indicated that surface topography characterization, particularly using S_sc_ and S_dq_, could improve the prediction of the remaining service life of wet-clutch friction materials. Groove geometry is another important surface-design factor and varies according to clutch application and operating requirements. Bao et al. solved the actual wear amount of different groove types (i.e., waffle grooves, three-way parallel grooves and two-way grooves) based on the Archard adhesive model and SAE#2 testing rig [74]. Yu et al. further showed, using a micro-contact numerical model, that reducing the groove area decreased wear but increased carbon-black accumulation [57].

#### 2.2.4. Wear Prediction

Repeated engagement cycles progressively degrade the mechanical integrity of friction materials. Thermal deterioration, carbonization, and elevated contact stresses can subsequently accelerate wear progression. To support condition assessment and maintenance planning, Ma et al. developed a wear prediction method for clutch friction components [42]. The method evaluated the current health state and future service performance of friction components based on material properties and operating parameters [42]. This capability allowed operators to adjust actual operating conditions in a timely manner, enabling conditional maintenance of clutches and extending service life. Li et al. developed a computational procedure for friction-lining wear prediction that successfully reproduced the experimentally observed two-stage wear behavior [41]. Furthermore, Li et al. performed model validation and uncertainty analysis for wet clutch wear prediction, emphasizing the importance of considering parameter variability and experimental uncertainty in durability assessment [43]. In addition, Zhang et al. presented a support vector machine regression (SVMR) model to predict running-in wear volume with field surface topography parameters and working conditions as the input variables [75]. The model retained satisfactory predictive performance despite the limited training dataset. The representative wear prediction approaches reviewed above are further compared in Table 5 in terms of their modeling methods, input information, prediction targets, and main characteristics.

### 2.3. Thermal Load

**Figure 5:**
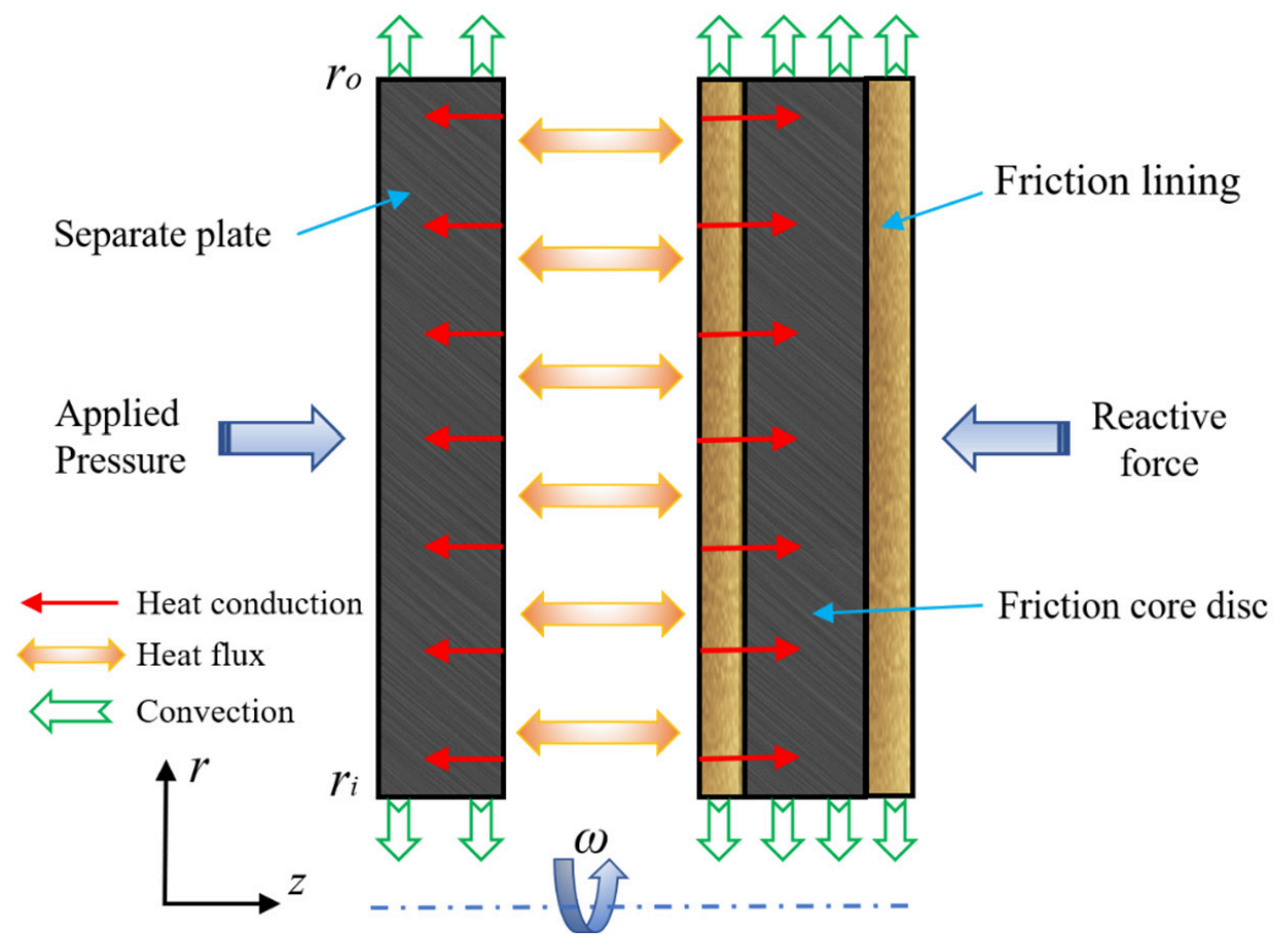
Thermal characteristics of friction components [40,76].

Time-varying thermal loading is a characteristic feature of clutch operation throughout its service life [40,76], as shown in Figure 5. Early studies on wet clutch thermal behavior established the foundation for understanding transient temperature evolution, heat partition, and thermal instability during engagement [77]. Subsequent numerical and experimental studies further examined transient thermal stresses [78,79], hot-spot initiation [80], and thermoelastic instability during wet-clutch engagement [45], providing important foundations for understanding thermally induced damage.

During engagement, frictional power can reach tens of kilowatts over a short time interval. The severe thermal load accelerates thermal damage accumulation in friction components, thereby degrading clutch performance and potentially leading to safety issues [81]. Accurately characterizing heat generation, conduction, and dissipation therefore remains a key challenge in clutch thermal analysis and monitoring.

#### 2.3.1. Heat Distribution

Friction enables torque transmission in the clutch but simultaneously generates the thermal load responsible for interface heating. The temperature field provides a direct representation of heat distribution within clutch friction components and has therefore been a long-standing focus of thermal analysis. With advances in computational capability, finite-element-based multi-physics modeling has become a major approach for investigating the thermo-mechanical behavior of clutch friction components. Earlier numerical studies by Zagrodzki investigated transient temperature fields and thermal stresses in multi-disc wet clutches, demonstrating the importance of thermomechanical coupling in predicting thermal damage and structural instability of friction components [78,79]. Zhao et al. investigated the thermomechanical behavior of carbon-carbon composite based on a transient temperature-displacement coupled analysis and facilitated the conceptual design of a multi-disc clutch system [82]. Zhang et al. subsequently incorporated the lubricant flow field into a coupled Abaqus–STAR-CCM+ model and showed that fluid cooling reduced the thermal load [83]. In practice, non-uniform interfacial contact can produce pronounced spatial variations in the temperature field.

The non-uniform temperature distribution may further induce thermal deformation and contact pressure redistribution, which can trigger thermoelastic instability (TEI) and hot spot formation. Jang and Khonsari developed a theoretical model to investigate hot spot initiation in wet clutches by considering the thermoelastic coupling behavior of friction materials and separator plates, revealing the influence of material properties and geometric parameters on thermal instability [80]. Yu et al. constructed a simplified pressure transmission model of the finite thickness solid to investigate the effect of concentrated reactive force generated by outer circlip on the temperature field [84].

#### 2.3.2. Heat Conduction

The heat partition coefficient (HPC) is a key parameter governing heat distribution between the contacting friction components. It quantifies the fraction of frictional heat transferred into each contacting body and is therefore central to predicting their transient thermal responses. Early studies commonly employed simplified assumptions, including semi-infinite bodies, perfect interfacial contact, and a constant HPC [85,86,87]. In a related brake-disc study, Méresse et al. identified local frictional heat fluxes using measured temperatures and a two-dimensional inverse heat-conduction model [88]. Subsequent studies therefore focused on determining more representative steady-state or time-varying HPC values. Xiong et al. proposed an equivalent method for a single-material contact body [89]. The method was used to calculate steady-state HPC over different thickness ratios, showing that the resulting value depended primarily on material density, heat capacity, and thickness ratio. Carmona et al. employed a reduced-order adjoint method for brake-disc heat-flux identification, reporting an average reconstruction error of 4.5% with a delay of 7 s [90]. Although demonstrated for a braking system, this approach provides a useful reference for computationally efficient thermal-state estimation.

#### 2.3.3. Heat Dissipation

Effective heat-dissipation measures are essential for mitigating the severe thermal loads experienced during clutch engagement. Lubricating oil flow provides the primary cooling mechanism for wet clutches. Using a thermal-resistance-network model, Ma et al. evaluated clutch temperature differences under different lubricant flow rates and found that further increases in flow produced only limited cooling benefits beyond a certain level [81]. The influence of material properties on heat transfer is also worth considering. Li et al. reported that lower skeletal density and specific heat capacity reduced the predicted engagement temperature under the investigated conditions [86]. Groove design also influences heat dissipation [74] by providing pathways for wear-particle removal and lubricant transport, thereby reducing surface contamination and enhancing local cooling.

Groove geometry influences the thermal and tribological behavior of friction systems. Li et al. investigated the effects of different groove patterns on the temperature-dependent wear of wet-clutch friction materials using thermohydrodynamic analysis [72]. In a related study, Abdullah and Schlattmann examined the influence of groove area ratio on the thermoelastic behavior of a single-disc dry clutch using finite-element analysis [91]. Although these studies involve different clutch configurations, they collectively highlight the importance of considering groove geometry in thermal and contact analyses.

Existing thermal models differ in their physical resolution, validation evidence, computational requirements, and suitability for online estimation. Table 6 compares representative studies using common reporting fields, including model formulation, validation conditions, reported prediction error, computational performance, and potential DT application. These fields provide a consistent basis for reviewing the available evidence, although direct numerical ranking is not appropriate because the studies consider different clutch configurations, thermal quantities, and validation conditions.

The comparison highlights a distinction between models suitable for online temperature estimation and those primarily supporting offline thermal analysis. Reduced-order thermal networks offer relatively efficient representations of overall heat transfer, although their spatial resolution is limited [81,92]. Among the reviewed approaches, Groetsch et al. explicitly demonstrated real-time temperature calculation and prediction using a validated parametric model [92]. Morris et al. reduced the computational burden of coupled thermal and hydrodynamic analysis to minutes, which is valuable for rapid design evaluation but does not establish real-time execution [93]. Detailed thermo-mechanical and coupled-field models provide information on local temperature gradients, contact effects, and thermal stresses [78,79,83,84], whereas dynamic heat-flux partitioning can improve temperature prediction under the investigated sliding conditions [94]. For a damage-oriented DT, these findings support combining offline model calibration with computationally efficient online temperature estimation. Nevertheless, computational latency and predictive accuracy must be evaluated under the target clutch’s operating conditions before an integrated real-time application can be established.

### 2.4. Dynamic Response

The dynamic response of a multi-disc clutch is influenced by the coupled effects of friction characteristics, structural parameters, thermal effects, and hydraulic actuation [40,76,95,96]. Variations in these factors during engagement may further alter the dynamic behavior of individual friction pairs and affect torque-transfer characteristics and engagement stability [40,76,96,97]. Therefore, dynamic responses such as friction torque and engagement judder provide important information for clutch condition assessment and health management [16,98,99].

#### 2.4.1. Friction Torque

Friction-torque models differ in physical fidelity, computational cost, and adaptability to operating conditions. Simplified isothermal and lumped-parameter models offer computationally efficient torque estimation, but their accuracy depends strongly on the prescribed friction coefficient [100]. Deur et al. developed and experimentally validated a dynamic wet-clutch engagement model incorporating fluid-film and actuator dynamics, demonstrating the importance of transient lubrication effects in clutch-response prediction [96]. Dynamic friction models can further represent nonlinear friction behavior and degradation-related changes [95,101], whereas coupled lubrication-contact models capture viscous and asperity-contact torques, temperature effects, and interactions among friction pairs [97,102]. However, these additional physical processes increase model complexity, parameter requirements, and computational demands.

For real-time DT applications, calibrated reduced-order models are promising candidates, while detailed multiphysics models are more suitable for offline analysis and calibration. Hybrid approaches offer another option for handling changing operating conditions. Wu et al. combined data-driven friction-coefficient estimation with a mechanistic torque model and reported a prediction error of 7.0% in clutch bench tests [103]. Nevertheless, model accuracy under untested conditions and full online computational performance require further evaluation. Therefore, model selection should consider prediction accuracy, computational efficiency, numerical stability during engagement transitions, and robustness to changes in pressure, speed, temperature, and friction characteristics.

#### 2.4.2. Engagement Judder

Clutch judder during engagement is an abnormal dynamic phenomenon that may accelerate damage accumulation and should be mitigated [98]. It not only degrades ride comfort but may also accelerate irreversible damage to friction components. Similar friction-induced vibration phenomena have been extensively studied in automotive friction systems. Jacobsson investigated the relationship between friction characteristics and vibration instability, demonstrating that friction variation and system dynamics play essential roles in judder generation [104].

Yu et al. developed a coupled thermo-dynamic model and showed that increasing temperature intensified hot judder [76]. To improve the robustness of judder prediction, Hao et al. developed an uncertain hybrid model combined with a hybrid perturbation vertex method [105]. Li et al. identified negative damping associated with a negative friction gradient as a major mechanism underlying clutch judder based on a nonlinear multi-body dynamic model considering stick-slip characteristics [106]. The same study further indicated that increasing the torsional stiffness or static COF could alleviate this phenomenon. The nonlinear friction-induced vibration mechanism was further analyzed by Ostermeyer, who revealed the influence of friction characteristics and system parameters on instability behavior [107].

#### 2.4.3. Control Strategy

Clutch-control strategies have evolved from conventional feedback regulation toward model-based and adaptive approaches. Li et al. developed a model predictive controller incorporating resistance-torque estimation and clutch-wear compensation [108], while Chen et al. proposed a self-learning fuzzy controller considering clutch wear [109]. Kim et al. used online estimates of friction characteristics and actuator parameters to improve engagement-torque control [110]. Yahagi et al. further demonstrated online PID gain adaptation for clutch slip control through vehicle experiments [111]. More recently, Zhang et al. integrated physical and digital clutch models with sliding-mode pressure control, achieving reductions of 46.6% in starting jerk and 1.1% in slipping friction work compared with PID control under the investigated hardware-in-the-loop conditions [112]. These studies demonstrate the benefits of feedback control and virtual-state estimation, although most do not explicitly address irreversible damage evolution.

For damage-oriented DT control, the operational feedback path can be organized around sensing, virtual-state estimation, parameter updating, and hydraulic-pressure regulation. Rotational-speed sensors on the driving and driven shafts provide the relative sliding speed, while hydraulic-pressure, torque, and temperature measurements characterize the actuation, transmitted load, and thermal response, respectively (Section 5.1). After synchronization and preprocessing, these measurements can be incorporated into a reduced-order virtual clutch model to estimate transient torque, sliding speed, and thermal states. Residuals between measured and predicted responses may support the updating of identifiable parameters, such as the effective friction coefficient and hydraulic-actuator gain or response characteristics [110,112]. In contrast, irreversible wear states require additional calibration using independent measurements or periodic inspections, since transient torque and temperature responses alone cannot uniquely determine physical wear.

Based on the updated virtual model, a supervisory controller could determine a target hydraulic-pressure profile to regulate clutch engagement while satisfying prescribed pressure, sliding-speed, and thermal constraints. The corresponding command would be executed through the electrohydraulic actuator, and subsequent measurements would be used to evaluate the response and update the virtual states. Control improvement should be assessed under comparable operating conditions using quantitative indicators such as sliding-speed tracking error, torque overshoot, engagement time, peak jerk, accumulated slipping friction work, and maximum temperature. The improvements reported by Zhang et al. [112] provide evidence for DT-assisted clutch-pressure control under the investigated hardware-in-the-loop conditions. However, these results do not establish an experimentally validated damage-aware SEDT control loop. In particular, state feedback, parameter recalibration, and higher-level evolution of damage models or knowledge-based rules remain distinct functions, and their integrated implementation requires further validation.

### 2.5. Failure

**Figure 6:**
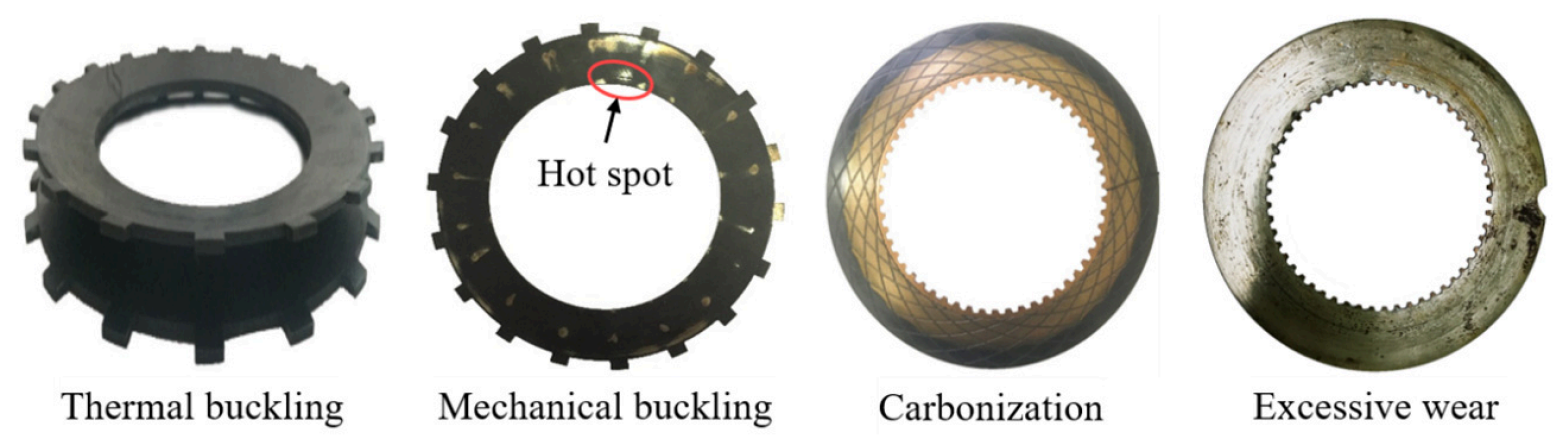
Common failure modes of friction components [48].

Failure represents the ultimate state reached when accumulated damage exceeds a critical threshold and results in the loss of functional or load-carrying capability. From the perspective of damage evolution, the major damage mechanisms of friction components can be broadly classified into thermally induced instability, mechanically induced structural instability, and fatigue-related material damage. Thermoelastic instability (TEI) may cause localized hot spots and severe thermal gradients; excessive thermal or mechanical stresses may induce buckling deformation of separator plates; and repeated thermo-mechanical loading may initiate and propagate fatigue cracks. In addition, long-term wear and material degradation can interact with these mechanisms and accelerate the transition from damage accumulation to functional failure. Representative failure manifestations, including buckling, hot spots, carbonization, cracking, and excessive wear [48], are shown in Figure 6.

Understanding and diagnosing damage mechanisms from friction-wear, thermal, and dynamic responses has therefore become a major focus of clutch research, with the aim of reducing failure risk and supporting health management. However, many existing studies remain focused on damage mechanisms and mitigation strategies at the level of individual friction components [113].

#### 2.5.1. Thermoelastic Instability and Hot Spotting

Thermoelastic instability (TEI) can induce localized hot spots and severe thermal gradients during clutch engagement, potentially accelerating material deterioration and increasing failure risk under severe operating conditions [45,114]. Early investigations of automotive friction systems systematically classified different forms of hot spotting and demonstrated that severe localized thermal loading can cause permanent material deterioration and significantly reduce the durability of friction components [115].

For wet multi-disc clutches, TEI may occur when the relative sliding speed exceeds a critical value [116]. Zagrodzki and Truncone investigated hot-spot generation during short-term engagement through numerical simulations and experimental tests. Their results demonstrated that friction-material properties and disc geometry strongly influence the onset and evolution of thermoelastic instability, and that typical clutch operating speeds may exceed the critical speed for hot-spot formation [45]. From a stability perspective, Zagrodzki further showed that friction clutches operating above the critical sliding speed can enter an unstable thermoelastic regime, in which small perturbations of contact pressure and temperature are progressively amplified [117].

To characterize hot-spot initiation associated with TEI, Hirano et al. proposed a cumulative-energy-based index, Qs1 [118]. In addition, thermal buckling is closely related to the in-plane stress of friction components. Using a curved-beam model, Xiong et al. calculated the critical in-plane bending moment and combined it with thermal-stress analysis to predict friction-component buckling [46]. Cui et al. further showed, using a transient thermal-deformation model, that deformation magnitude was strongly influenced by the number and locations of constraints [113].

#### 2.5.2. Mechanical Buckling

Buckling deformation of thin separator plates can arise from different loading mechanisms. From a thermo-mechanical perspective, Audebert et al. demonstrated that thermal excursions may generate residual in-plane bending moments sufficient to trigger either axisymmetric coning or non-axisymmetric buckling of automatic-transmission clutch plates [47]. In addition to thermally induced buckling, mechanical torque transmitted through the spline constraints can also produce circumferential instability.

Mechanical buckling can occur when the torque transmitted through the outer spline teeth exceeds the corresponding critical buckling torque. Yu et al. developed a mechanical-buckling model for a separator plate and investigated the relationship between resisting torque and periodic bright spots [48]. The results identified excessive resisting torque as a major cause of high-order circumferential buckling of the separator plate. By using the Mindlin plate theory and the axisymmetric flatness conditions, Bagheri et al. found that the critical buckling torque and its shape depended on the boundary conditions, radius ratio and thickness ratio [119]. Beyond mechanical modeling, recent studies have explored vibration-based identification of clutch-plate buckling. Feng et al. proposed an improved Hilbert–Huang transform method incorporating wavelet packet decomposition and Hilbert spectral entropy to classify buckling severity in wet multi-disc clutches [120]. Their experimental results demonstrated the potential of time–frequency vibration features for distinguishing different buckling conditions. However, the method primarily addresses fault classification rather than continuous estimation of plate deformation or subsequent damage evolution.

Existing wet-clutch buckling studies primarily provide deterministic critical conditions based on thermo-mechanical and plate-stability analyses [46,47,48,119]. For clutch-specific probabilistic assessment, uncertainties in transmitted spline torque, contact and thermal loads, separator-plate geometry, material properties, and boundary conditions could be introduced into a calibrated buckling model. The resulting instability probability can be expressed as the probability that the applied load exceeds the corresponding critical limit. For mechanical buckling, this may be represented by *P*(*T*_spline_ ≥ *T*_cr_), where *T*_spline_ and *T*_cr_ denote the transmitted spline torque and critical buckling torque, respectively. The stochastic thermal-buckling approach reported by Lal et al. [121] provides a methodological reference for uncertainty propagation, but its material properties, boundary conditions, and numerical results cannot be directly transferred to wet multi-disc clutches. Clutch-specific calibration and independent validation using measured loading conditions, separator-plate deformation, and buckling observations are therefore required. Since buckling may occur abruptly, instability probability and time to first threshold exceedance are more appropriate prognostic quantities than a continuously accumulated damage index.

#### 2.5.3. Fatigue Crack

Severe thermo-mechanical coupling can generate macroscopic hot spots that promote fatigue-crack initiation and propagation. Similar damage mechanisms have been extensively investigated in high-energy friction braking systems. Dufrénoy and Weichert combined damage observations with thermo-mechanical modeling and showed that repeated thermal loading produces cyclic stresses that promote the initiation and propagation of macroscopic cracks on friction surfaces [53]. Experimental observations by Panier et al. further demonstrated that macroscopic hot spots generate severe thermal gradients and cyclic thermo-mechanical stresses, providing favorable conditions for low-cycle thermal fatigue and crack initiation [54]. Kasem et al. further correlated local hot-spot temperatures with subsurface material damage, including plastic deformation, microcrack formation, and phase transformation, demonstrating that measurable thermal responses can serve as indirect indicators of irreversible damage evolution [122]. In the railway brake discs investigated by Yang et al. [55], dominant cracks were observed to propagate mainly in the radial direction. Whether similar propagation patterns occur in wet-clutch separator plates requires further investigation. Combining experimental tests with numerical simulations, Li et al. investigated the effect of braking energy on fatigue-crack evolution and found a strong correlation between crack depth and the residual tensile-stress distribution [123]. Wu et al. analyzed thermal-fatigue cracking and used crack-propagation behavior to assess brake-disc safety under repeated emergency braking [124].

Studies of railway brake discs have established relationships among cyclic thermo-mechanical loading, fatigue-crack propagation, and fatigue life [53,55,123,124,125]. However, these findings provide methodological references rather than direct validation for wet multi-disc clutches. A clutch-specific probabilistic crack-growth model would require the thermal-stress history of the separator plate, an initial crack-size distribution, material-specific crack-growth parameters, and a defined critical crack size. Bayesian updating, as demonstrated for an aircraft structure by Karandikar et al. [126], could incorporate independently calibrated crack measurements to update uncertain model parameters and predict distributions of failure probability and remaining useful life. Routine torque, temperature, or vibration measurements would provide indirect information unless their relationships with crack size were separately validated. Differences in component geometry, spline constraints, loading spectra, material properties, and lubrication conditions prevent direct transfer of crack-growth parameters or predicted failure probabilities from brake discs and aircraft structures. Consequently, application to wet multi-disc clutches requires dedicated crack-growth measurements, parameter calibration, and independent experimental validation.

### 2.6. Existing Limitations

Existing studies provide complementary approaches for characterizing clutch wear, thermal instability, and structural damage [41,43,45,46,48]. However, translating these mechanisms into online health assessment requires distinguishing measurable operating signals from damage states that must be estimated through models. Table 7 summarizes their potential relationships, including candidate monitoring inputs, damage-related state variables, and corresponding DT functions. The mapping represents a literature-informed synthesis rather than a fully validated online damage-monitoring system.

Based on the reviewed studies and the damage-to-DT mapping summarized in Table 7, three major limitations can be identified.

1. Develop high-fidelity system-level models. Many existing models simplify multi-disc clutches as axisymmetric single-friction-pair systems, thereby neglecting differences and interactions among individual friction components and limiting system-level understanding of clutch behavior. High-fidelity system-level models are therefore required to capture component-level differences and their coupled effects throughout clutch operation.
2. Capture time-varying operating states. Although vibration-based methods have demonstrated the feasibility of classifying wet-clutch buckling conditions [120], continuous estimation of internal damage states remains challenging. In related tribological research, Gutierrez et al. demonstrated that acoustic emission signals combined with machine learning could estimate the coefficient of friction in sliding steel contacts [127]. However, this evidence does not establish acoustic-emission-based wear-depth monitoring in wet multi-disc clutches. Further investigation is needed to determine how vibration, acoustic emission, torque, and thermal measurements can be jointly interpreted to estimate underlying clutch damage states rather than only observable performance indicators.
3. Realize online damage-aware health management. Current clutch health-management approaches are predominantly offline or reactive, resulting in delayed intervention and potentially allowing irreversible damage to accumulate before maintenance is performed. There is therefore a clear need for online damage diagnosis, prognosis, and health-management strategies that can support timely intervention and extend clutch service life.

Addressing these limitations requires validated links between measurable operating responses, mechanism-specific damage states, and DT-based assessment functions, motivating the DT approaches reviewed in the following section.

## 3. Development of DT

### 3.1. Origin

The early concept of DT can be traced back to 2003, when Grieves introduced it in the context of product lifecycle management (PLM) [128]. The concept was subsequently referred to as the mirrored spaces model (MSM) [28]. This early formulation established the basic three-part DT structure comprising the physical space, virtual space, and the data and information connections between them. The resulting physical–virtual interaction and bidirectional information exchange provided a basis for real-time monitoring, model updating, and optimization [129]. However, the concept received limited attention at that time because the sensing, computing, and communication technologies required for practical implementation were not yet sufficiently mature. In 2011, the U.S. Air Force Research Laboratory further advanced the DT concept for aircraft structural-life prediction by integrating high-fidelity computational models with detailed geometric, material, and operational information [29]. By incorporating flight-history data, such models can estimate the evolving structural state and damage condition of aircraft components to support life prediction and health management. In 2012, NASA and the U.S. Air Force Research Laboratory further formalized the DT paradigm for future aerospace vehicles operating under demanding and long-duration service conditions [130]. They defined DT as a high-fidelity representation of an as-built aircraft or system that integrates multiphysics, multiscale, and probabilistic modeling. By integrating high-fidelity physical models, sensor updates, and operational history, the DT can continuously reflect the condition and predicted service life of the corresponding physical system [30]. This paradigm extended DT from a conceptual representation toward a lifecycle-oriented framework for the development, certification, operation, and maintenance of complex engineering systems [131]. These developments marked an important transition toward the broader recognition and application of DT in engineering research.

### 3.2. Related Work

Digital twin (DT) architectures have evolved from basic physical–virtual representations toward integrated frameworks incorporating data, services, and continuous interaction. Tao et al. developed a five-dimensional DT model comprising physical entities, virtual models, services, DT data, and connections, and explored its application to equipment prognostics and health management [132,133]. Subsequent studies investigated product-oriented DT architectures [134], enabling technologies [19], evolutionary DT concepts [34], and probabilistic damage prognosis within DT frameworks [135]. These developments provide the theoretical foundation for damage-oriented DT applications. However, adapting them to wet multi-disc clutches requires explicit consideration of coupled frictional, thermal, and mechanical damage mechanisms, together with their observability and prognostic requirements.

**Figure 7:**
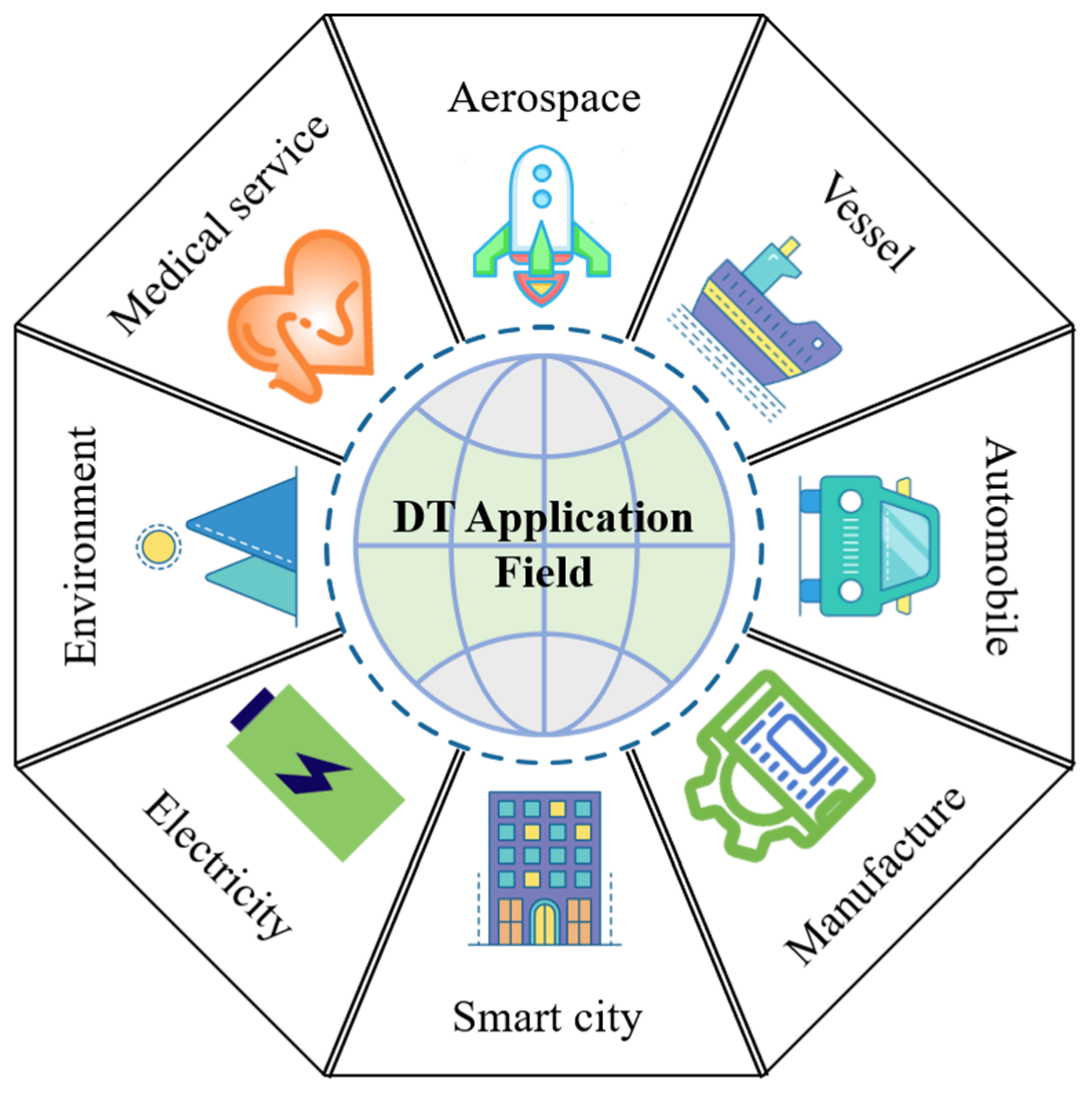
Application fields of digital twin technology (adapted and reorganized from Qi et al. [19] with reference to related studies).

Because of its capability for cyber–physical integration, DT has attracted extensive attention in both academia and industry, with applications reported in aerospace [29,135], marine systems [136], automotive engineering [137,138], healthcare [139,140], and smart cities [36,141]. The representative application fields of DT technology are summarized in Figure 7 based on Qi et al. [19] and the related studies reviewed in this section.

1. Design and Manufacturing.
  **The digital twin workshop (DTW) provides a framework for improving product quality and manufacturing adaptability under changing production requirements, consistent with the development of intelligent manufacturing [28,142].** Individualized and adaptive optimization of manufacturing processes has consequently become an important DT research direction. Ma et al. presented an AI-enhanced digital twin shop-floor framework and detailed its interaction and implementation mechanisms [143]. To support personalized design, Zhang et al. proposed a DT-based approach for the design and multi-objective optimization of a hollow-glass production line [144]. Lin et al. further proposed the concept of an evolutionary digital twin (EDT) to improve flexibility in intelligent industrial product development [34].
2. Equipment health management.
  **Beyond product design and manufacturing, increasing attention has been directed toward in-service performance monitoring and health management to improve operational safety and reliability [138].** Prognostics and health management (PHM) has become an important research area in mechanical engineering, and its integration with DT has attracted increasing interest in both academia and industry [145,146]. For online monitoring, Song et al. developed a DT-based performance prediction model for an electro-optical detection system together with a system-level performance indicator [33]. Seshadri et al. proposed a DT-based method for real-time assessment of aircraft damage size and residual load-carrying capacity [147]. For fault diagnosis, Cai developed a DT-based virtual machine-tool framework that uses historical digital traces to support diagnosis and prognosis when faults occur in the physical machine tool [148]. Wang et al. developed a modified DT model with a parameter-sensitivity-based updating scheme for rotating machinery, enabling fault diagnosis and degradation analysis [13]. For damage prognosis, Li et al. developed a probabilistic model for the diagnosis and prediction of fatigue-crack growth in aircraft wings [135]. Magargle et al. demonstrated DT-enabled predictive maintenance for an automotive braking system by integrating Modelica-based system modeling with finite-element analysis [138]. For life assessment, Aivaliotis et al. evaluated the health condition of industrial robots and predicted their remaining useful life using historical operational data [15]. Zakrajsek et al. developed a DT-based aircraft-tire wear model to monitor tire health under non-ideal touchdown conditions [149].
  Recent advances in physics-informed machine learning (PIML) have provided additional opportunities to combine mechanistic knowledge with data-driven diagnosis and prognosis. Deng et al. systematically reviewed the integration of physical knowledge into machine learning for prognostics and health management, particularly under limited-data conditions [150]. Li et al. further classified physics-informed RUL prediction approaches into physical-model–data fusion, stochastic degradation modeling, and PIML-based methods [151]. These approaches offer potential strategies for incorporating wear laws, thermo-mechanical constraints, and uncertainty information into data-driven clutch health assessment. Nevertheless, their effectiveness for wet-clutch damage estimation requires application-specific validation.
  Recent DT studies of transmission-related components also provide useful methodological references. Zhou et al. developed a vibration-based DT for online gearbox assessment by integrating dynamic-load estimation, vibration-based model calibration, and fatigue-damage evaluation [152]. Feng et al. proposed a gear-wear DT incorporating a spatially varying Archard wear model and measurement-based updating of wear coefficients [153]. For rolling bearings, Peng et al. combined physics-generated vibration data with adaptive domain alignment to improve fault diagnosis under non-stationary operating conditions [154]. These studies demonstrate distinct capabilities in damage modeling, parameter updating, and cross-domain diagnosis. However, their transfer to wet multi-disc clutches requires further consideration of lubricated friction interfaces, coupled thermal effects, and the observability of internal damage states. A structured comparison of representative approaches in terms of their application objects, damage-state representation, updating mechanisms, and limitations for clutch health management is provided in Table 8.
3. Environment and life service.
  **Beyond industrial applications, DT technology has also been extended to environmental management, healthcare, and smart-city services.** In the context of Industry 4.0, Sin et al. proposed a standardized and modular DT-based solution for smart energy management [155]. Coraddu et al. developed a data-driven DT for vessels to estimate speed loss and additional fuel consumption caused by marine fouling [136]. In healthcare, Joshi et al. applied a DT-driven precision-nutrition strategy to support diabetes management [140]. Smart-city systems also provide opportunities to improve resource utilization and support more sustainable urban management [36]. Austin et al. proposed a smart-city DT architecture supporting semantic representation and discussed its implementation methods and associated challenges [156].

## 4. Conceptual SEDT Architecture

**Figure 8:**
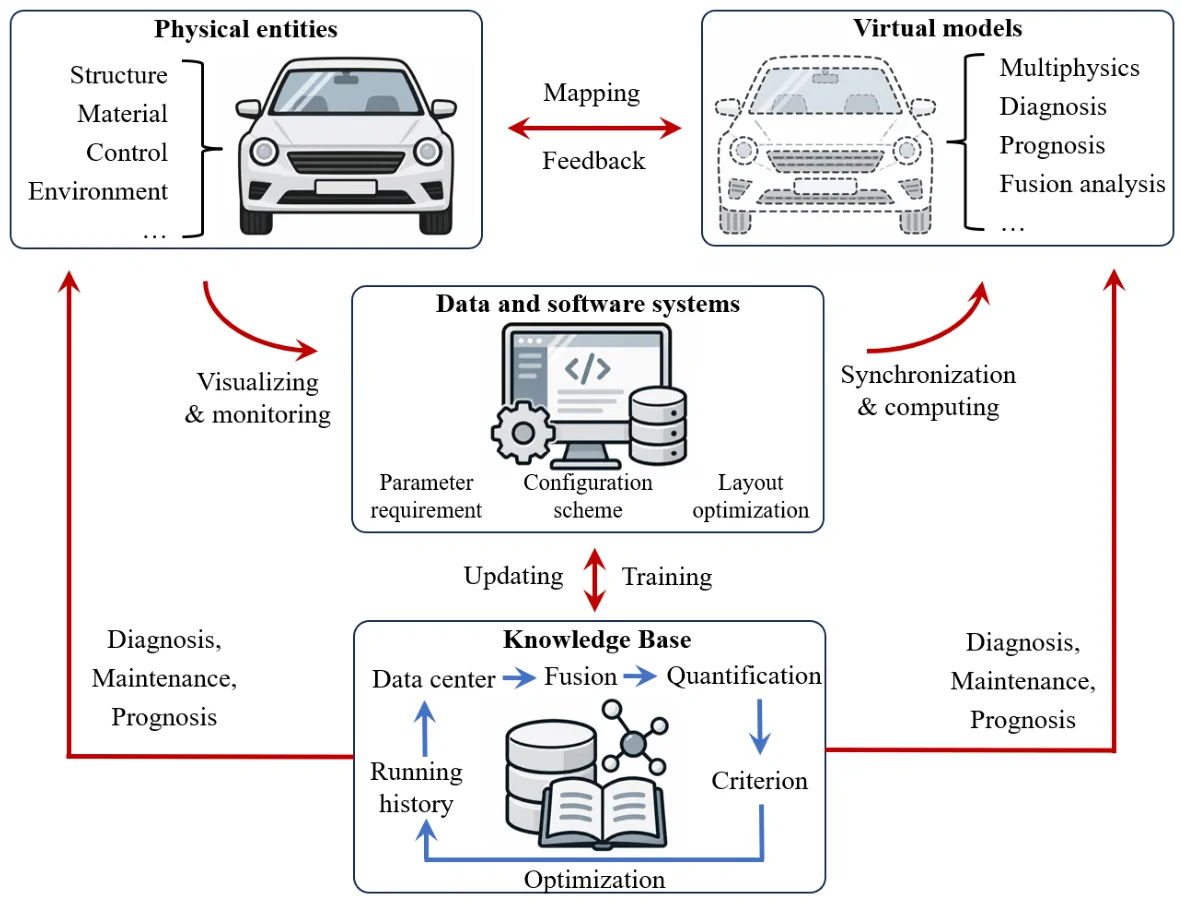
Conceptual SEDT architecture synthesized from established digital twin and evolutionary digital twin approaches (authors’ synthesis based on Refs. [19,34,132,133]).

The proposed SEDT architecture is a literature-informed synthesis of established DT concepts, rather than a fundamentally new DT structure. It organizes physical entities, virtual models, data and software systems, and a knowledge base into four functional components (Figure 8). These components broadly correspond to the physical entity, virtual model, data and connections, and service functions of existing five-dimensional DT frameworks [132,133]. Related studies have already demonstrated DT-based health management [132,135,138] and learning-enabled model evolution [34]. Therefore, the inclusion of a knowledge base or model-updating capability alone does not constitute the originality of the present framework.

The contribution of this review lies in a damage-oriented synthesis tailored to multi-disc clutches rather than the development of a new general-purpose DT architecture. The physical–virtual structure and data connections are drawn from established DT frameworks [19,132,133], while learning-enabled model adaptation [34] and individual damage-modeling and prognostic methods [41,43,45,48,135] originate from previous studies. Building on these foundations, this review integrates the relationships among clutch damage mechanisms, measurable operating responses, damage-state estimation, uncertainty assessment, and health-management decisions into a unified conceptual framework. The distinctions between existing approaches and the proposed SEDT are summarized in Table 8. Higher-level evolution of damage-model representations and knowledge-based decision rules is identified as a prospective capability rather than a demonstrated implementation. Although individual methods in the reviewed literature have been experimentally investigated, the numerical example in Section 5.5 demonstrates only synthetic wear-state prediction and parameter updating, together with the computational cost of a simplified scalar update. It does not validate wear-prediction accuracy against experimental data or the integrated SEDT framework. Therefore, the proposed architecture and its self-evolving functions still require system-level experimental validation.

### 4.1. Physical Entity

A complete physical object constitutes the foundation of DT modeling and serves as the physical-space counterpart of the virtual model [19,157]. The physical entity comprises the real-world components and subsystems that possess measurable geometric, material, structural, and operational attributes. It encompasses the multidisciplinary characteristics of the target system, including geometry, material properties, assembly constraints, operating environment, and dynamic behavior. In addition, the physical entity should be equipped with sufficient sensing and communication capabilities to support real-time acquisition and transmission of multi-source heterogeneous data. Because system behavior evolves during degradation, the physical entity should provide sufficient actuation and control interfaces to support adaptive operational adjustment within the SEDT framework.

### 4.2. Virtual Model

The virtual model provides a high-fidelity representation of the physical entity in virtual space. It extends beyond geometric replication by representing material properties, operating conditions, physical interactions, and dynamic behavior. The virtual model should provide a high-fidelity and dynamically updated representation of the physical entity by integrating geometry, material properties, operating conditions, and damage-related behavior [13,26,35]. Such a dynamically updated virtual representation provides two primary benefits: (1) damage-related abnormalities can be identified and localized during operation; (2) the evolution of physical parameters, operating characteristics, and damage states can be tracked throughout the system lifecycle. Accordingly, several factors should be considered when constructing the virtual model:

1. The high-fidelity virtual model should represent not only the properties of individual components but also the coupled constraints and interactions among them.
2. Given computational constraints, model fidelity should be allocated according to the relative importance of different components and physical processes.
3. The virtual model should incorporate degradation- and damage-related state variables to reflect the evolving in-service condition of the physical entity.

### 4.3. Data and Software System

The data and software system provides the information infrastructure connecting the physical and virtual spaces and comprises sensing devices, data-acquisition hardware, communication interfaces, and software platforms. Real-time data acquisition, heterogeneous data integration, and bidirectional information interaction are fundamental to maintaining consistency between the physical and virtual spaces [17,19,24].

Effective data twinning requires coordinated consideration of data acquisition and data interaction. For data acquisition, the required state variables and damage-related indicators should first be identified. Appropriate sensors and data-acquisition devices should then be configured to ensure reliable measurement and transmission of these variables. The sensing configuration can subsequently be refined as operating conditions, damage mechanisms, and monitoring requirements evolve. For data interaction, the software platform should provide standardized interfaces that enable communication among heterogeneous sensors, devices, databases, and virtual models. Representative software tools for DT implementation include ANSYS for physics-based modeling and simulation, Simulink for multidomain physical modeling, MATLAB for data-driven diagnosis and prognosis, and Python-based tools for data processing and fusion [19]. These platforms are illustrative implementation options rather than a list of software used to generate results in this review. The numerical demonstration in Section 5.5 was performed using Python 3.13.5; any future SEDT implementation would require a software configuration appropriate to its modeling, data-interface, and computational requirements.

After preprocessing, synchronization, and integration, multi-source heterogeneous data can be mapped to the virtual model to support process and performance twinning. This bidirectional data flow provides the basis for physical–virtual synchronization, model updating, and closed-loop decision support.

### 4.4. Knowledge Base

The knowledge base provides structured information and reasoning support for the conceptual SEDT. Its functions can be organized into three interconnected layers: knowledge representation, probabilistic inference, and decision support. The representation layer may employ an ontology and knowledge graph to associate clutch components, operating conditions, damage mechanisms, monitoring indicators, and maintenance actions, supplemented by expert-defined rules and experimentally calibrated thresholds [158,159]. The inference layer combines monitoring features with virtual-model outputs to update damage-state probabilities and degradation predictions [135,160]. The decision layer uses these estimates to evaluate failure risk and select maintenance actions according to predefined objectives [161,162].

Information exchange among the SEDT components follows a closed-loop process. The data system supplies synchronized measurements, while the virtual model provides predicted responses and damage-related variables. Their discrepancies support state estimation and parameter calibration, and the knowledge base returns updated health estimates, diagnostic conclusions, and maintenance recommendations. Importantly, routine data accumulation or parameter updating does not by itself constitute self-evolution. In the proposed framework, higher-level evolution requires evidence-supported revisions of damage-model representations or knowledge-based rules, followed by independent validation before their adoption. Such functionality remains a prospective capability rather than a demonstrated implementation.

## 5. Literature-Informed Synthesis of SEDT for Damage-Oriented Health Management of Multi-Disc Clutches

Existing studies provide complementary foundations for damage-oriented health management of multi-disc clutches. Physics-based wear models have been developed to characterize friction-material loss and its dependence on operating conditions [41,43], while thermo-mechanical analyses have investigated hot-spot formation and structural instability under severe engagement conditions [45,46]. In addition, stochastic degradation models and vibration-based diagnostic methods have been applied to clutch RUL prediction and buckling identification [10,120]. Recent DT studies of gearboxes and gear wear have further demonstrated the potential of measurement-informed model calibration and damage assessment [152,153]. However, these approaches differ in their physical interpretability, measurable state variables, computational requirements, and degree of experimental validation. Their individual capabilities do not necessarily establish an integrated framework for tracking multiple damage mechanisms in wet multi-disc clutches.

**Figure 9:**
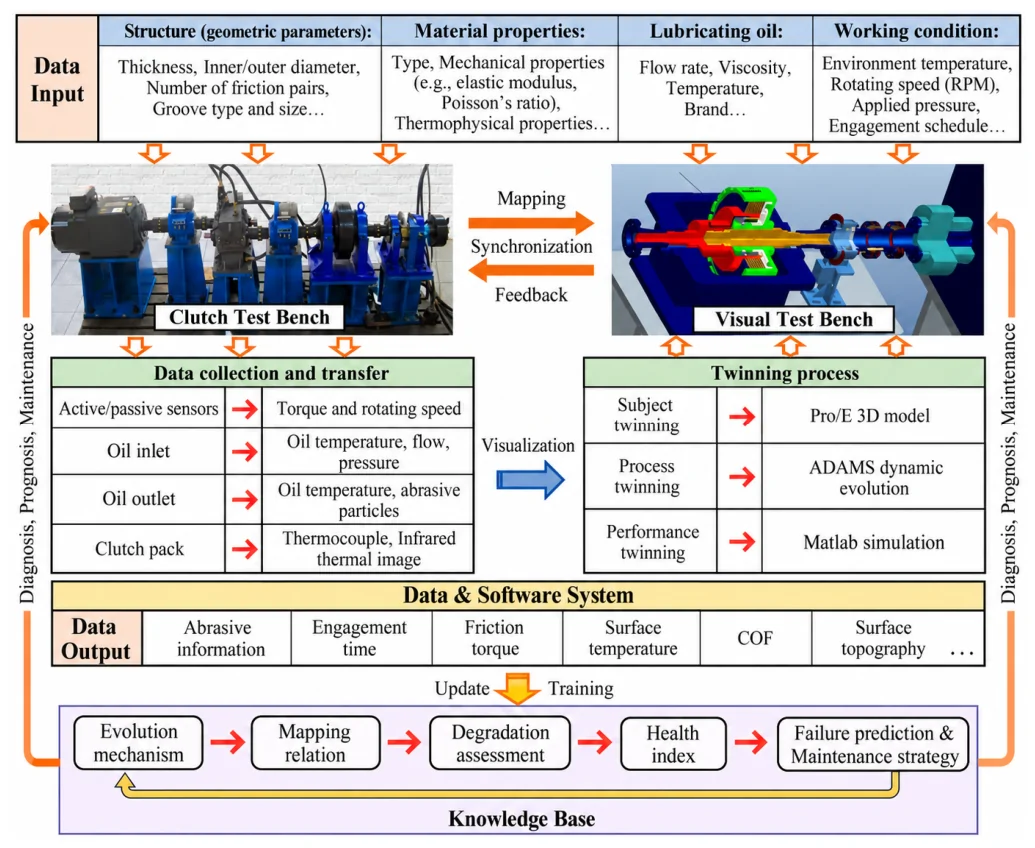
Conceptual SEDT framework for damage-oriented health management of multi-disc clutches (authors’ synthesis based on Refs. [19,34,41,45,48,132,133]).

Based on these findings, this section critically examines the sensing, modeling, data processing, and health-management functions required for damage-oriented clutch monitoring. The representative methods and their limitations are compared to identify the challenges associated with integrating physical damage evolution into a DT. Figure 9 presents a literature-informed synthesis of the proposed SEDT framework, comprising a physical entity, virtual model, data and software system, and knowledge base. The framework remains conceptual, and the associated implementation and validation requirements are discussed in the following subsections.

### 5.1. Physical Entity: Damage-Aware Clutch System

**Figure 10:**
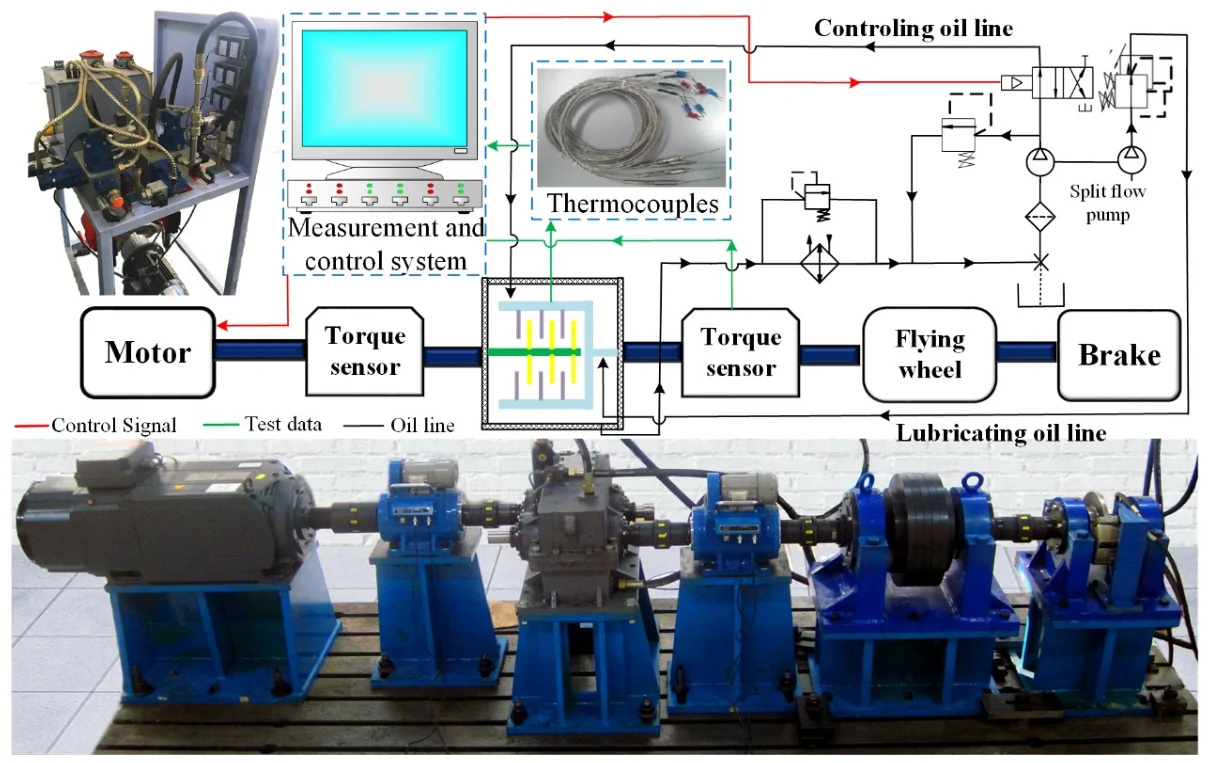
Schematic of the self-developed laboratory clutch test bench [49].

**Figure 11:**
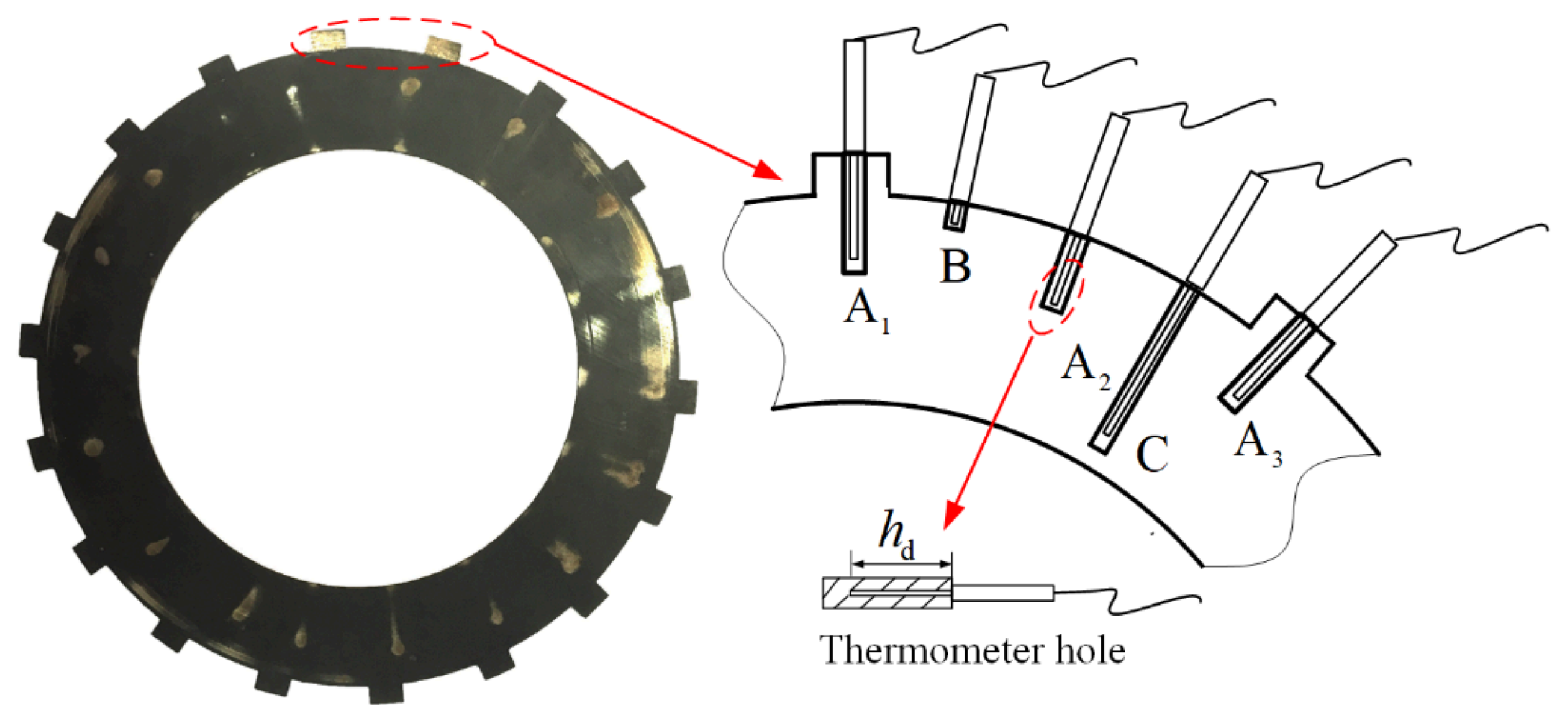
Temperature measurement layout of a separator plate [49].

The self-developed laboratory clutch test bench reported in Ref. [49] is used as an illustrative physical platform to demonstrate how the proposed conceptual SEDT framework could be implemented. The reference is intended to support the experimental configuration and temperature-measurement layout shown in Figure 10 and Figure 11, rather than the SEDT architecture itself.

Existing wet-clutch test rigs provide experimental foundations for torque and thermal-response monitoring [49], while dedicated durability tests have demonstrated the possibility of estimating clutch-pack wear from piston-displacement measurements [163]. For the proposed damage-oriented monitoring system, rotational-speed sensors should be arranged on the driving and driven sides to determine relative sliding speed. Torque measurement can characterize transmitted load, while hydraulic-pressure sensors near the engagement-piston circuit provide information for estimating the applied contact force. Temperature measurements at the lubricant inlet and selected separator-plate locations can support thermal-state assessment, with radially and circumferentially distributed measurement points providing additional information on temperature nonuniformity.

Wear-state calibration may be supported by piston-displacement measurements or periodic friction-lining inspections, subject to appropriate compensation and calibration [163]. Transient operating signals should be acquired at rates sufficient to resolve engagement dynamics, whereas wear inspection and parameter correction may occur at longer intervals. Additional wear-debris, vibration, or acoustic sensors may improve damage observability, but their installation and effectiveness require separate verification. The sensing configuration therefore represents a prospective implementation scheme rather than an experimentally validated arrangement of the complete SEDT.

### 5.2. Virtual Model: Damage-Aware Digital Twin of the Clutch

In the conceptual SEDT framework, the virtual model serves as the primary representation for damage-state estimation and evolution modeling. High-fidelity three-dimensional models of clutch components can first be established using CAD tools to represent geometry, assembly constraints, and material distributions. Multi-physics simulation tools can subsequently be used to model engagement dynamics, friction-torque evolution, thermo-mechanical coupling, and other damage-related processes.

The damage-related variables incorporated into the virtual model should be distinguished from directly observed performance indicators. Following the mapping in Table 7, measured pressure, speed, torque, and temperature can first be used to characterize clutch operating conditions and estimate difficult-to-measure internal responses through physics-based models [40,48,76]. Damage-evolution models can then relate these operating states to underlying damage variables, such as wear depth, thermal deterioration, or structural deformation [41,43,46,48]. Model parameters and estimated states may be updated using available observations, whereas independent wear measurements, deformation tests, or post-test inspections are required to assess the reliability of damage estimates. This provides a prospective pathway from operational monitoring to damage-state estimation within the conceptual SEDT framework.

Through real-time data feedback and model updating, discrepancies between simulated responses and physical measurements can be progressively reduced. Accordingly, the virtual model is intended to reproduce both operational behavior and the evolving damage state of the physical clutch throughout its service life.

Physics-informed machine learning may provide an intermediate modeling approach between high-fidelity multiphysics simulation and purely data-driven prediction [150,151]. Within the proposed SEDT, physical relationships such as wear accumulation laws and thermal energy balances could be incorporated as model constraints or used to generate physically consistent training data. Measurement-based parameter updating, as demonstrated in related gear-wear DT research [153], may further support adaptation under changing operating conditions. However, these approaches do not eliminate the need for damage-state observability, independently calibrated measurements, or validation of prediction accuracy and computational efficiency in wet-clutch applications.

**Figure 12:**
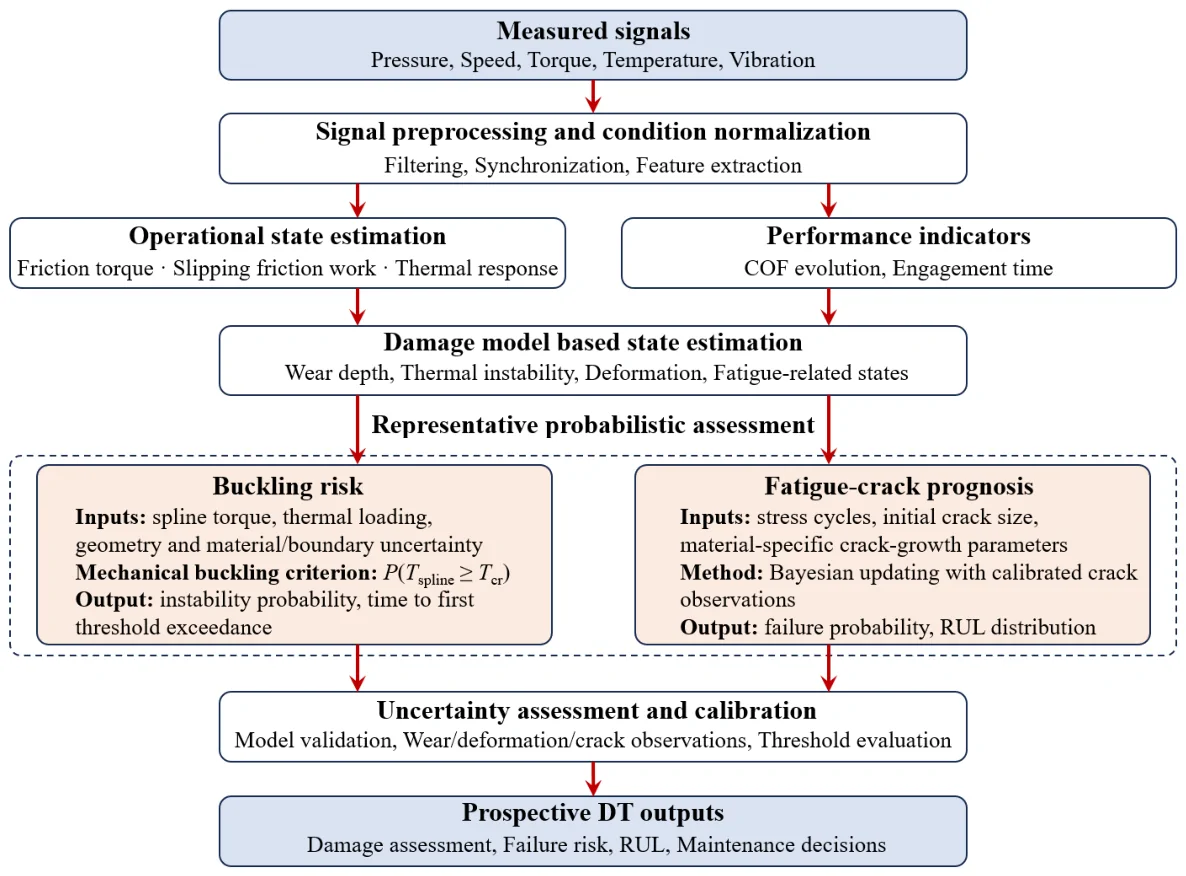
Conceptual workflow for clutch damage-state estimation and probabilistic health assessment (authors’ synthesis based on Refs. [13,41,43,45,48,121,125,126,160,164]).

Figure 12 illustrates the conceptual workflow from measurable clutch signals to damage-state estimation, uncertainty assessment, and health-management outputs. Two representative probabilistic pathways are included: mechanical-buckling risk assessment based on the torque transmitted through separator-plate splines and the corresponding critical buckling torque, and fatigue-crack prognosis considering cyclic stresses, initial crack size, and uncertain crack-growth parameters. The former aims to estimate instability probability and time to first threshold exceedance, whereas the latter supports the prediction of failure probability and remaining useful life distributions. Both pathways require clutch-specific model calibration and independent validation using deformation measurements or calibrated crack observations. Importantly, operating signals and performance indicators alone cannot uniquely determine irreversible damage, and these probabilistic pathways represent prospective modeling approaches rather than experimentally validated SEDT functions [41,43,48,121,125,126,160].

As summarized in Table 2, Table 3, Table 4 and Table 7 and Figure 12, wear, thermally induced damage, structural instability, and fatigue cracking represent different physical damage states that require distinct monitoring indicators and estimation methods. Within the proposed SEDT, the virtual model is intended to provide damage-state estimates, associated uncertainty, and information for prognosis and risk assessment. However, internal deformation and crack evolution cannot generally be inferred uniquely from indirect monitoring signals and require calibrated models and independent validation.

### 5.3. Data and Software System: Multi-Source Damage Feature Fusion

The data and software system provides the information infrastructure connecting the physical entity, virtual model, and knowledge base. It is responsible for the acquisition, transmission, processing, storage, and management of multi-source operational and damage-related data. Data preprocessing, heterogeneous information fusion, and physical–virtual synchronization are widely recognized as enabling functions of DT systems [17,19,24]. Accordingly, in the proposed conceptual SEDT, raw signals acquired from the physical clutch can be processed through filtering, synchronization, normalization, and feature extraction before being mapped to the corresponding virtual-model states. As discussed in Section 2.4.3, the data system can also provide an interface between virtual-state estimation and physical clutch control [108,110,112]. Updated state estimates and model predictions may inform actuator commands, while subsequent sensor measurements provide feedback for evaluating the response and correcting the model. Such an operational feedback loop should be distinguished from the higher-level damage-model and knowledge evolution envisaged in the conceptual SEDT.

Existing monitoring studies indicate that different measurement channels provide different levels of information about clutch damage. Torque, rotational speed, and temperature measurements primarily characterize operating and thermal responses, while vibration features have been used to classify buckling conditions in wet multi-disc clutches [49,120]. In related sliding-contact studies, acoustic emission signals have been used to estimate friction characteristics, although such measurements do not directly establish physical wear depth [127]. Dedicated piston-displacement measurements offer a more direct means of monitoring clutch-pack wear under calibrated test conditions [163]. These findings highlight the need to distinguish measured operating variables, indirect damage-sensitive features, and independently verified damage observations. For the proposed SEDT, time-synchronized sensor data could be combined with model predictions, whereas periodic wear inspections and other independently calibrated observations would be needed to correct the corresponding damage states. The effectiveness of this data-fusion strategy remains dependent on sensor accuracy, observation-model validity, and experimental verification.

### 5.4. Knowledge Base: Damage Diagnosis, Prognosis, and Health Management

**Figure 13:**
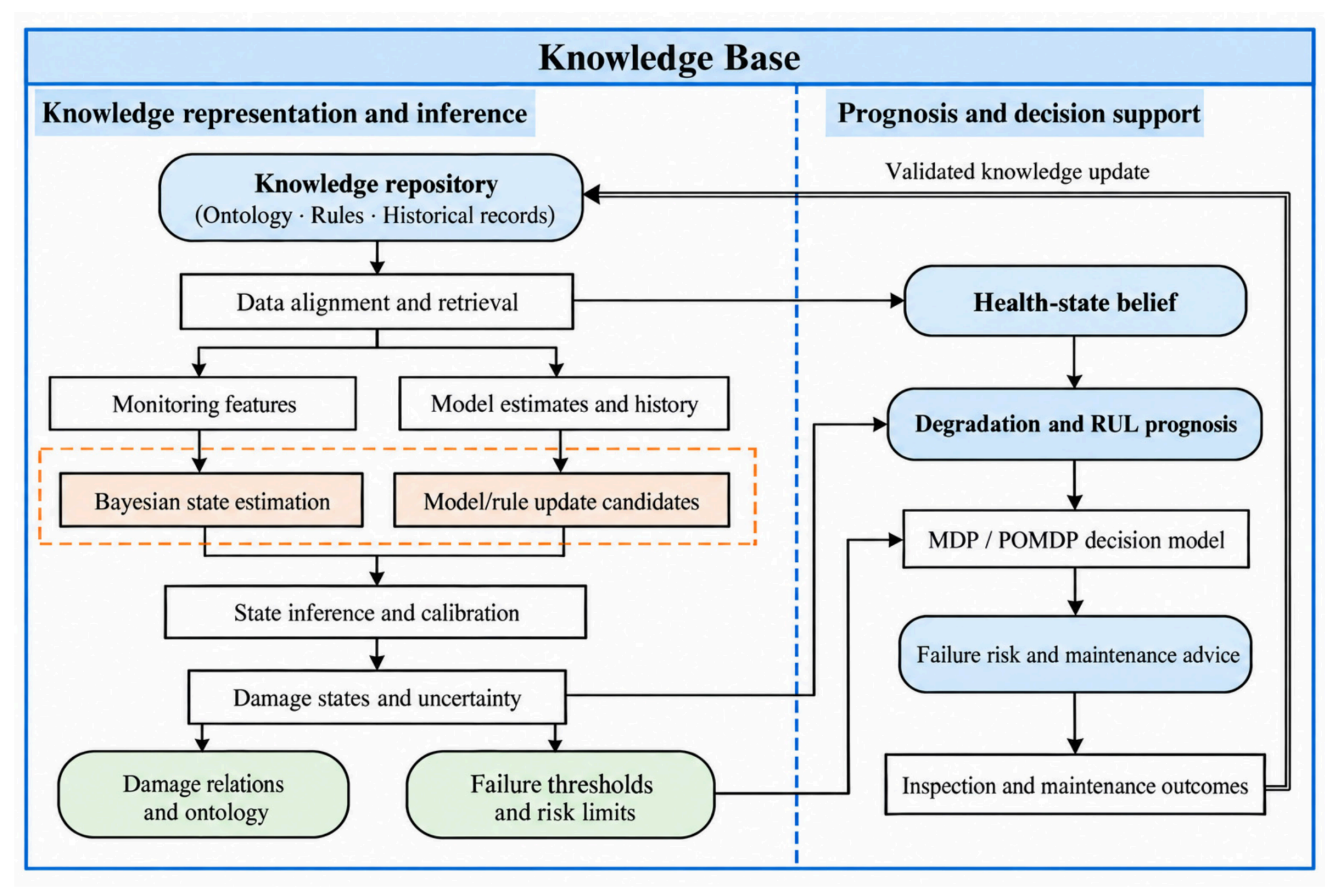
Conceptual knowledge-base framework for damage diagnosis, prognosis, and maintenance decision support (authors’ synthesis based on Refs. [135,158,159,160,161,162,164,165,166,167]).

The knowledge base serves as the reasoning and decision-support core of the conceptual SEDT framework. Existing PHM studies have established condition diagnosis, degradation prognosis, RUL prediction, and maintenance decision-making as interconnected elements of equipment health management [12,14,16]. For clutch systems, wear- and degradation-based RUL prediction has also been investigated using dynamic and stochastic degradation models [10,56]. Building on these studies, a conceptual damage-oriented knowledge-base framework is synthesized in Figure 13.

The proposed knowledge-base functions can be supported by established probabilistic diagnosis, degradation prognosis, and maintenance optimization methods. Bayesian state-space models provide a means of estimating latent damage or degradation states from noisy observations [135,160], while ensembles of probabilistic classifiers can combine fault-diagnosis results and estimate the probabilities of candidate fault classes [165]. Stochastic degradation models can then be used to predict failure probabilities and RUL distributions [164,166]. For sequential maintenance decisions, MDP methods are applicable when the decision state is treated as observable, whereas POMDP methods explicitly account for uncertainty in hidden states [162,167].

For a representative wear-oriented clutch application, the decision state space can be defined as S = {normal, degraded, critical, failed}, with state boundaries determined from system-specific damage and functional criteria. The observation space may include operating-condition-normalized COF, torque, temperature, and calibrated inspection measurements. Candidate actions are A = {continue operation, inspect, preventive maintenance, corrective maintenance}. The reward function can be expressed as *r*(*s*, *a*) = −[*c*(*a*) + *c*_down_ *d*(*s*, *a*) + *c*_fail_*P*_fail_(*s*, *a*)], where *c*(*a*) denotes the action-dependent cost, *d*(*s*, *a*) represents the expected downtime, and *P*_fail_(*s*, *a*) is the conditional failure probability. The objective is to maximize the expected discounted reward. State transitions can be derived from calibrated degradation models, while Bayesian filtering updates the belief over possible states when direct damage observations are unavailable.

Computational feasibility remains a major constraint for online Bayesian inference and POMDP optimization. If m interacting damage variables are each discretized into q levels, the joint state space may contain q^m^ states, making exact inference and decision optimization increasingly expensive as damage dimensionality grows. Practical alternatives include offline model calibration, reduced-order state representations, particle or sequential filtering, and approximate policy optimization [167,168]. Their computational costs depend on the number of states or particles, the forward-model complexity, and the decision horizon. Consequently, inference accuracy, computational latency, and robustness must be evaluated for the target clutch system before real-time implementation can be claimed.

Through closed-loop interaction among the physical entity, virtual model, data and software system, and knowledge base, the SEDT framework is intended to support online damage diagnosis, prognosis, and health-management decision making, thereby linking damage-mechanism research with prospective in-service applications. The approaches reviewed above differ in their damage representation, model-updating capabilities, and prognostic functions. Table 9 provides an evidence-based comparison of representative methods and their limitations for damage-oriented clutch health management.

As summarized in Table 9, existing studies have established important but distinct capabilities in physical damage modeling, fault identification, degradation prognosis, and measurement-informed model updating. However, the available evidence does not yet establish an experimentally validated wet-clutch DT that jointly tracks multiple irreversible damage states and integrates their evolution with uncertainty-aware prognosis and maintenance decisions. The proposed SEDT is therefore presented as a conceptual integration of these complementary approaches rather than a demonstrated improvement in predictive accuracy or computational performance. Its practical value will depend on independently validated damage observations, appropriate model fidelity, reliable updating mechanisms, and system-level experimental verification.

The complete SEDT framework has not yet been implemented and experimentally validated as an integrated closed-loop system. The clutch test bench and measurement configuration shown in Figure 10 and Figure 11 provide a potential experimental platform for future implementation, whereas the individual damage mechanisms, modeling approaches, and DT technologies underlying the framework are supported by the literature reviewed in the preceding sections. Future work should validate the accuracy of damage-state estimation, model updating, RUL prediction, and maintenance decision making under representative clutch operating conditions.

Several limitations of the conceptual SEDT framework should be acknowledged. First, the complete physical–virtual closed loop has not yet been experimentally implemented and validated. Second, some damage state variables, such as internal thermal damage and stiffness degradation, cannot be directly measured and therefore require reliable estimation or data-fusion methods. Third, the computational cost of high-fidelity multi-physics models may conflict with the real-time requirements of online model updating. Moreover, damage thresholds and model parameters are dependent on friction materials, clutch geometry, lubrication conditions, and operating profiles. Therefore, the accuracy, robustness, and transferability of the framework across different clutch systems remain to be systematically validated. Future validation should assess sensor reliability, damage-state observability, end-to-end computational latency, and predictive accuracy using independent clutch test data, rather than evaluating model execution or physical–virtual communication in isolation.

### 5.5. Illustrative Application Scenario: Wear Prediction Under Varying Loads

To illustrate a potential implementation of the conceptual SEDT, a wear-prediction scenario under varying clutch loads is considered. An Archard-type model can provide a computationally efficient description of wear accumulation based on contact pressure and sliding distance [56]. For more detailed analysis, the thermohydrodynamic wear model developed by Li et al. incorporates thermal degradation and mechanical effects and reproduces the two-stage wear behavior of paper-based clutch friction materials [41]. Its subsequent validation and uncertainty analysis further highlight the importance of uncertain model inputs [43]. These approaches can serve different functions within a prospective DT, with simplified models supporting sequential state estimation and detailed models providing offline calibration and physical interpretation.

**Figure 14:**
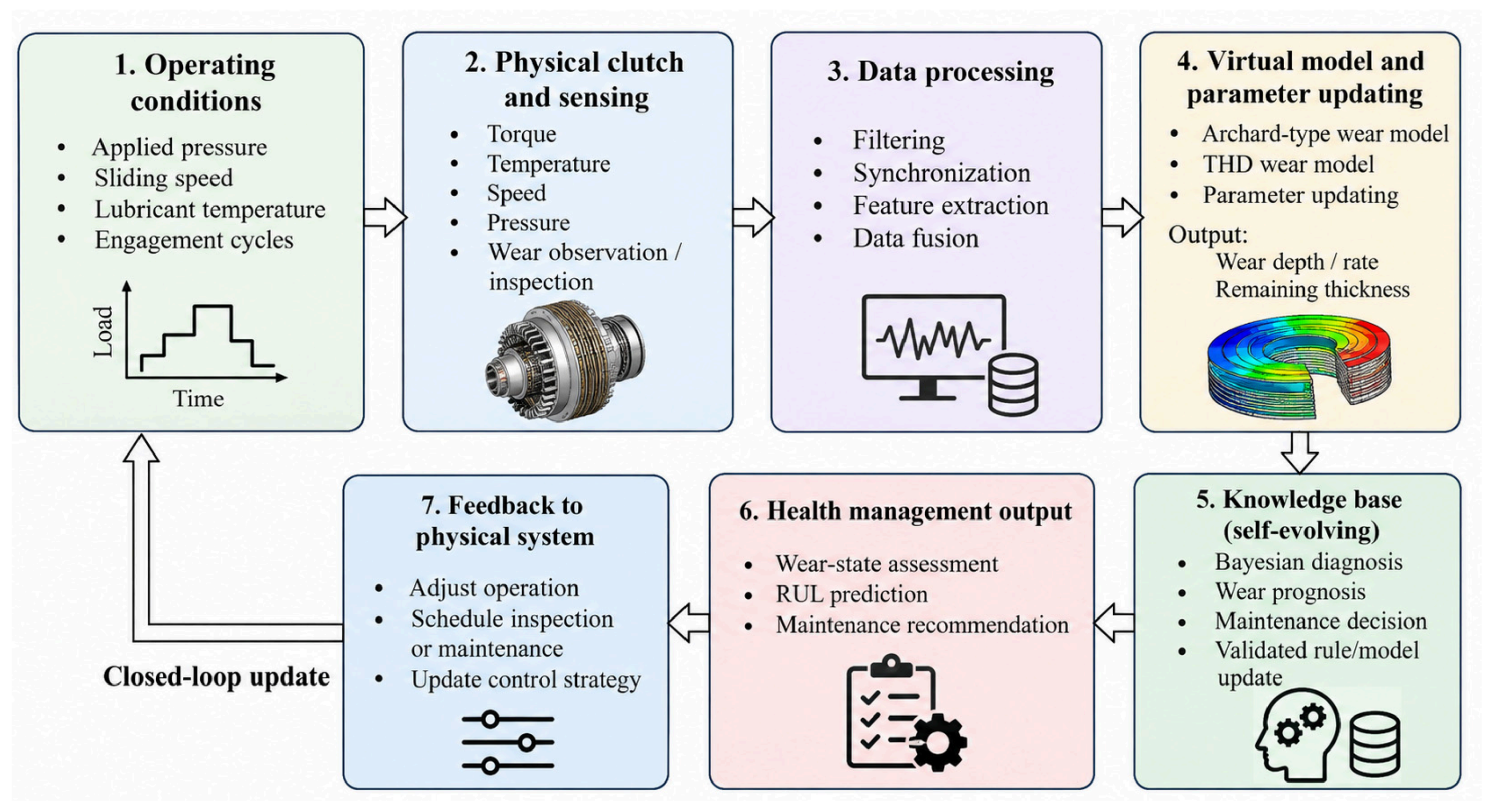
Illustrative workflow for wear-state prediction and parameter updating under varying clutch loads (authors’ synthesis based on Refs. [41,43,163]).

The required operating inputs include hydraulic pressure, rotational speeds, temperature, and engagement duration. Effective contact pressure and sliding speed must be derived from these measurements using calibrated clutch geometry and mechanical relationships. Wear observability depends on the available instrumentation. Lingesten et al. demonstrated continuous monitoring of clutch-pack wear using piston-displacement measurements on a dedicated test rig [163]. Such measurements, after appropriate compensation, could provide observations for wear-state correction and parameter updating. In contrast, COF, torque, and temperature alone do not uniquely identify wear depth because they are affected by multiple operating and material factors. The proposed workflow for wear-state prediction, observation-based parameter updating, and prospective health-management decisions is illustrated in Figure 14.

To provide a reproducible illustration of the proposed workflow, a simplified Archard-type wear model is adopted. The cumulative wear depth after each operating block is calculated as

(1) $$h_{j}=h_{j-1}+\kappa N_{j}p_{j}v_{j}\Delta t_{j}$$

where $h_{j}$ is the cumulative wear depth (μm) after block j; κ is the effective wear coefficient (μm/(MPa·m)); $N_{j}$ is the number of clutch engagements; $p_{j}$ is the representative effective contact pressure (MPa); $v_{j}$ is the representative sliding speed (m/s); and $\Delta t_{j}$ is the effective sliding duration per engagement (s). The model assumes constant representative pressure and speed within each operating block.

For sequential parameter updating, the cumulative loading term for block *j* is defined as

(2) $$S_{j}=N_{j}p_{j}v_{j}\Delta t_{j}$$

Using the wear observation from the preceding block and the previously estimated wear coefficient, the predicted cumulative wear depth before the next update is

(3) $${\hat{h}}_{j|j-1}=z_{j-1}+{\hat{\kappa }}_{j-1}S_{j}$$

where $z_{j-1}$ denotes the latest available wear-depth observation and ${\hat{\kappa }}_{j-1}$ is the estimated wear coefficient after the preceding update. Assuming zero initial wear, a constant effective wear coefficient, and periodically available calibrated wear observations, a simplified cumulative parameter estimate can be obtained as

(4) $${\hat{\kappa }}_{j}=\frac{z_{j}}{\sum_{i=1}^{j}S_{i}}$$

This updating relationship is used only to demonstrate the numerical procedure. In practice, measurement noise, parameter variability, and model uncertainty would require more robust estimation methods, such as weighted least squares or Bayesian filtering.

A fully synthetic example is constructed using three successive operating blocks, each comprising 10,000 clutch engagements with an effective sliding duration of 0.5 s per engagement. The representative contact pressures are 1.0, 1.5, and 2.0 MPa, while the corresponding sliding speeds are 7, 10, and 13 m/s. The nominal lubricant temperature is assumed to remain at 100 °C and is not explicitly incorporated into this simplified wear calculation. An effective wear coefficient of 1.2 × 10^−4^ μm/(MPa·m) is assumed to generate the synthetic observations, whereas the estimator starts with an initial coefficient of 1.0 × 10^−4^ μm/(MPa·m). The generated cumulative wear values are rounded to the nearest micrometer to obtain observations of 4, 13, and 29 μm. Table 10 summarizes the wear predictions and sequential parameter updates.

The assumed pressure and sliding-speed conditions fall within the experimental ranges investigated by Gong et al. for copper-based wet-clutch friction materials [44]. Nevertheless, the effective wear coefficient, engagement duration, and wear observations in Table 10 are synthetic assumptions rather than parameters calibrated from that study. As shown in Table 10, the initial model predicts a cumulative wear depth of 3.50 μm after the first operating block. The corresponding synthetic observation of 4 μm is used to update the effective wear coefficient. With the updated parameter, the predicted cumulative wear depths before subsequent observations are 12.57 and 28.36 μm, compared with synthetic observations of 13 and 29 μm, respectively. These results illustrate how changes in operating conditions and periodically available wear observations can be incorporated into sequential wear-state prediction and model-parameter correction.

Nevertheless, this example demonstrates only the numerical feasibility of the proposed information flow, rather than experimentally validated wear-prediction accuracy. The simplified model assumes a constant effective wear coefficient and does not reproduce the temperature-dependent, two-stage wear behavior described in previous wet-clutch studies [41,43]. Furthermore, direct wear observations may only be available from dedicated measurements or periodic inspections [163]. When relying solely on indirect signals such as COF, torque, and temperature, wear-depth estimation remains subject to observability limitations and model uncertainty. Consequently, practical SEDT implementation requires independently calibrated observation models and validation under representative variable-load conditions.

The computational feasibility of the illustrative wear-state update was further assessed using a CPU-based implementation. With an assumed state-update frequency of 20 Hz, the three operating blocks were represented by 300,000 scalar wear-state updates. The numerical test was conducted using Python 3.13.5 on an Intel Core i9-13900HX processor. The total execution time was approximately 0.047 s, with a 99th-percentile computation time of approximately 0.11 μs for an individual scalar wear-state update. The numerical predictions were consistent with the results presented in Table 10.

These results demonstrate the low computational cost of the simplified wear-state calculation but do not establish the real-time performance of the complete SEDT system. The benchmark excludes physical data acquisition, communication, signal processing, coupled multiphysics simulation, probabilistic inference, and maintenance control. Furthermore, the assumed update frequency does not represent a validated sampling requirement for transient clutch dynamics. Therefore, the feasibility of the integrated SEDT framework still requires experimental assessment of end-to-end latency, computational resources, damage-state estimation accuracy, and system robustness.

## 6. Opportunities and Challenges

The rapid development of DT reflects the growing demand for digitalization, intelligent operation, and cyber–physical integration across industrial systems. Continued advances in sensing, modeling, data analytics, and artificial intelligence provide new opportunities for SEDT development, while also introducing challenges related to standardization, computational efficiency, system integration, data security, scalability, and validation.

1. Cross-domain application opportunities: As reviewed in Section 3, DT concepts have already been explored in aerospace, marine systems, automotive engineering, healthcare, smart cities, and other engineering domains. These cross-domain developments provide opportunities to adapt and extend the proposed SEDT framework by incorporating domain-specific models, sensing strategies, and health-management requirements.
2. Integration with emerging technologies: The implementation of SEDT relies on the coordinated development of enabling technologies, including advanced sensing, big-data analytics, cloud and edge computing, artificial intelligence, machine learning, high-performance computing, and communication technologies. Integrating these technologies could improve the real-time capability, model-updating efficiency, and decision-support performance of future SEDT systems.
3. Standardization and policy support: The increasing industrial adoption of DT is also expected to promote the development of technical standards, implementation guidelines, and regulatory frameworks for interoperable and reliable DT systems.

Despite these opportunities, several challenges must be addressed before SEDT can be implemented as a reliable engineering tool.

1. Standardization and interoperability: The implementation of SEDT across different systems requires standardized terminology, data formats, communication interfaces, model-exchange protocols, and validation procedures while retaining sufficient flexibility for domain-specific requirements.
2. Computational efficiency and real-time data processing: However, high-fidelity multi-physics modeling, real-time data processing, and continuous physical–virtual synchronization impose substantial computational and communication demands. Future implementation will therefore require efficient reduced-order or surrogate models, advanced computing architectures, and reliable low-latency communication to balance model fidelity with real-time performance.
3. System integration, data security, and scalability: Practical SEDT implementation requires integration with existing automotive ECUs, onboard sensors, and communication networks through standardized interfaces. Continuous data exchange also raises concerns regarding data security, privacy, and access control. Moreover, fleet-wide deployment introduces additional challenges in data volume, communication bandwidth, and computational resources, which may require scalable edge–cloud architectures.

## 7. Conclusions and Future Work

Digital twin technology provides a promising paradigm for cyber–physical integration and lifecycle health management of complex engineering systems. Against this background, this review investigates how DT concepts can be integrated with clutch damage mechanisms and synthesizes a conceptual SEDT framework for damage-oriented monitoring and health management of multi-disc clutches. The specific contributions are as follows.

First, recent advances in multi-disc clutch research are systematically reviewed from the perspectives of friction-wear behavior, thermal load, dynamic response, and damage-related failure mechanisms. Second, a conceptual SEDT architecture comprising the physical entity, virtual model, data and software system, and knowledge base is synthesized to establish a structured link between clutch damage evolution and DT-enabled health management. Third, the prospective implementation of the SEDT framework is illustrated using an existing clutch test platform, with particular emphasis on damage-aware sensing, virtual modeling, multi-source data fusion, diagnosis, prognosis, and maintenance decision making. Overall, this review provides a systematic framework for connecting clutch damage-mechanism research with DT-enabled monitoring and health management and identifies key requirements for future implementation and validation.

Because the proposed SEDT framework is formulated at a functional level, it has the potential to be adapted to different multi-disc clutch configurations and related friction systems. Beyond multi-disc clutches, the functional SEDT architecture may also be extended to other transmission components, such as gears and bearings. The general closed-loop structure integrating the physical entity, virtual model, data system, and knowledge base can be retained, while component-specific damage indicators should be introduced, such as tooth wear or pitting for gears and vibration or defect indicators for bearings. However, such transfer requires system-specific adaptation and validation of the physical model, sensing strategy, damage indicators, model parameters, decision thresholds, and prognostic models.

Future work should therefore focus on integrated closed-loop implementation, experimental validation of damage-state estimation and RUL prediction, improvement of real-time model-updating efficiency, and evaluation of framework robustness and transferability across different clutch systems.

## Figures and Tables

**Table 1:** Overview of multi-disc clutch research.

Research Directions	Numerical Models	Main Studies	Approaches
Friction–wear	•Archard wear model •Hertz contact theory model •Gauss distribution law…	•Material •Coefficient of friction •Morphology •Wear prediction	◆ Test ▸SAE-II test bench ▸Pin-on disk tester… ◆ Software ▸Abaqus ▸Ansys ▸Star CCM+… ◆ Data processing ▸Fast Fourier transform ▸Explicit Euler ▸Monte Carlo statistical method… ◆ Algorithm ▸Self-learning fuzzy control ▸Adaptive sliding-mode control ▸Model predictive control…
Thermal load	•Multiphysics coupling mode •Thermal resistance network model…	•Heat distribution •Heat conduction •Heat dissipation	
Dynamic response	•Gauss distribution law •Random elastic contact model •Mixed uncertainty model •Thermal-dynamic model…	•Friction torque •Engagement judder •Control strategy	
Failure	•Curved beam model •Rayleigh–Ritz methods •Timoshenko beam theory •Mindlin plate model…	•Thermoelastic instability •Mechanical buckling •Fatigue crack	

**Table 2:** Classification and quantification of damage mechanisms in multi-disc clutches.

Damage Mechanism	Evolution Type	Typical Manifestation	Main Cause	Quantitative Indicators
Wear	Progressive	Material loss, surface deterioration	Repeated friction	Wear depth, wear volume, mass loss
Thermal/material degradation	Progressive	Carbonization, property deterioration	Repeated thermal loading	Temperature history, material-property change
Fatigue damage	Progressive	Crack initiation/ propagation	Cyclic thermo-mechanical loading	Crack length/depth, propagation rate
TEI-induced hot spotting	Rapid-onset	Hot spots	Thermoelastic feedback	Critical speed, hot-spot temperature
Thermal buckling	Rapid-onset	Coning/distortion	Severe thermal stress	Deformation/ buckling amplitude
Mechanical buckling	Rapid-onset	Circumferential deformation	Excessive mechanical load	Critical torque, deformation amplitude

Note: The classification reflects the dominant evolution time scale rather than a universal damage threshold. Transient instability does not necessarily imply irreversible material damage.

**Table 3:** Representative damage-related thresholds and critical conditions reported for wet clutches.

Ref.	Damage Mechanism	Reported Threshold or Boundary	Specific Context
[44]	Wear regime transition	Load- and speed-dependent transition boundaries	Copper-based material against alloy steel; tested at 1.0–3.0 MPa and 6.7–20.1 m/s
[45]	TEI and hot spotting	Critical sliding speed, dependent on clutch configuration	Wet multi-disc clutch; transient engagement and different friction-material/geometric configurations
[49]	Circumferential elastic deformation	Critical circumferential temperature difference: 2–3 °C	Separator plate in the investigated laboratory clutch configuration
[50]	Temperature-associated clutch failure	Temperature above 300 °C observed as necessary for failure	Three tested wet-clutch friction systems in limited-slip differential applications

Note: The reported thresholds and boundaries are specific to the investigated materials, configurations, and operating conditions. The numerical values should not be interpreted as universally applicable clutch failure limits.

**Table 4:** Representative interactions between progressive damage and rapid-onset instability in friction systems.

Initiating Mechanism	Affected Mechanism	Interaction Pathway	Evidence
Wear	TEI	Changes in material thickness and wear rate may either promote or suppress instability	[51]
TEI-induced hot spotting	Wear/material deterioration	Localized heating increases thermal degradation and wear susceptibility	[41,45]
Thermal buckling	Thermal and frictional response	Nonuniform contact increases local temperature and torque fluctuations	[46,52]
Repeated hot spotting	Fatigue cracking	Cyclic thermal stresses promote crack initiation and propagation	[53,54,55]

Note: The interaction pathways are condition-dependent and do not necessarily occur in every clutch system. Evidence for the hot-spotting–fatigue-cracking relationship is primarily derived from related brake-disc studies.

**Table 5:** Comparison of representative wear prediction methods for clutch friction materials.

Ref.	Method	Main Inputs	Prediction Target	Main Characteristics
[42]	Mathematical wear model	Oil pressure; sliding speed; engagement cycles	Wear amount	Experimentally parameterized; validated over 18,000 shifting cycles
[41]	THD-based thermal–mechanical wear model	Material/operating parameters; temperature; stress	Wear rate/ lining loss	Captures thermal and mechanical degradation and two-stage wear
[43]	Validated wear model with UQ	Uncertain model inputs	Temperature and wear evolution	Sensitivity analysis, Monte Carlo validation, and uncertainty quantification
[75]	SVMR	Surface-topography parameters; operating conditions	Running-in wear volume	Effective with small training datasets; MRMR-based feature selection

**Table 6:** Comparison of representative thermal models for wet multi-disc clutches in terms of validation, accuracy, computational performance, and online applicability.

Ref.	Model and Main Features	Validation Conditions	Reported Accuracy	Computational Performance	Potential Application
[81]	Thermal resistance network; lumped thermal model	Wet-clutch hydraulic system; different oil-flow conditions; experimental comparison	Quantitative error NR	Runtime NR	System-level thermal assessment; potential reduced-order use
[78,79]	Transient thermo-mechanical modeling	Multi-disc wet-clutch thermal and stress analysis	Standardized error NR	Runtime NR	Offline temperature and stress analysis
[83]	Coupled thermo-hydro-mechanical model	Wet-clutch temperature and stress simulations	Quantitative error NR	Runtime NR	Offline coupled-field analysis
[84]	Contact-pressure and thermal finite-element models	Wet multi-disc clutch; bench validation under creeping conditions	Quantitative error NR	Runtime NR	Offline local temperature and stress assessment
[92]	Parametric thermal network with temperature prediction	Automotive and industrial wet-clutch tests; continuous and non-steady slip	Typically <5 K temperature deviation	Real-time operation demonstrated; numerical latency NR	Online temperature estimation and thermal-load prediction
[93]	Simplified 1D thermal model with hydrodynamic interface effects	Wet multi-disc clutch; experimental temperature comparison and drive-cycle analysis	Quantitative error NR in the accessible summary	Runtime on the order of minutes	Rapid offline evaluation and design studies
[94]	Thermal model with dynamic heat-flux partition	Limited-slip clutch sliding-bench tests	<0.1 °C/s for temperature-rise rate; <3 °C for average temperature	Runtime NR	Temperature prediction and offline model calibration

Note: NR indicates that a comparable quantitative value was not established from the sources available for this comparison; it does not necessarily mean that the original study contains no such information. The reported errors correspond to different quantities and experimental conditions. Real-time applicability is identified only where supported by the cited study.

**Table 7:** Mapping of clutch damage mechanisms to monitoring signals, state variables, and prospective DT functions.

Damage Mechanism	Candidate Monitoring Signals	Estimated Damage-Related States	DT Functions	Supporting Refs.
Wear	Torque, pressure, sliding speed, temperature, wear debris	Wear depth, wear rate, remaining lining thickness	Wear-state estimation, RUL prediction	[41,43]
Thermal/ material degradation	Temperature history, thermal cycles	Thermal exposure, material deterioration	Thermal- damage risk assessment	[41,48]
TEI-induced hot spotting	Sliding speed, spatial temperature variation	Hot-spot severity, TEI stability margin	Instability monitoring and warning	[45,80]
Thermal buckling	Temperature distribution, thermal gradient	Buckling amplitude, critical-load margin	Thermal- instability assessment	[46,49]
Mechanical buckling	Torque, pressure, structural response	Deformation state, critical-torque margin	Structural- instability assessment	[48,119]
Fatigue damage	Thermal cycles, strain, vibration or acoustic signals	Crack size or damage probability	Fatigue prognosis and RUL assessment	[53,54,55]

Note: Monitoring signals are candidate inputs rather than direct measurements of irreversible damage. Damage-state estimation requires appropriate models and calibration. Several pathways, particularly online crack estimation, remain to be validated for wet multi-disc clutches.

**Table 8:** Comparison of representative DT frameworks and the proposed conceptual SEDT for damage-oriented clutch health management.

Approach	Main Contribution	Damage/Health Management Capability	Limitation Relative to This Review
Tao et al. [132,133]	Five-dimensional DT with data, services, and connections	General DT-enabled PHM	Not specific to coupled clutch damage mechanisms
Li et al. [135]	Dynamic Bayesian network for aircraft-wing DT	Probabilistic crack diagnosis and prognosis	Aircraft-specific structural damage
Magargle et al. [138]	Simulation-based automotive braking-system DT	Model-based thermal monitoring and predictive maintenance	Braking system; limited representation of clutch damage evolution
Lin et al. [34]	Evolutionary DT using learning and policy exploration	Learning-based model and behavior adaptation	Not focused on clutch wear or damage-state prognosis
Feng et al. [120]	Vibration-based buckling diagnosis for wet multi-disc clutches	Classification of clutch buckling severity	Lacks continuous damage-state estimation and DT integration
Zhou et al. [152]	Vibration-based gearbox DT with dynamic model calibration	Gearbox fatigue-damage assessment	Not specific to coupled clutch damage mechanisms
Feng et al. [153]	Gear-wear DT with Archard-based modeling and parameter updating	Wear-state monitoring and prediction	Not validated for wet-clutch wear and health management
Peng et al. [154]	Physics-driven bearing DT with adaptive domain alignment	Fault diagnosis under non-stationary conditions	Lacks continuous clutch damage prognosis and maintenance decisions
Conceptual SEDT (this review)	Integrates clutch damage mechanisms, monitoring, and PHM into a domain-oriented synthesis	Proposed damage-state estimation, uncertainty-aware prognosis, and knowledge-based decisions	Integrated implementation and self-evolution remain unvalidated

**Table 9:** Comparison of representative modeling approaches for clutch health management.

Approach	Main Basis	Online Updating	Damage Interpretability	Prognosis	Main Limitation
Physics-based wear models [41,43]	THD and wear evolution models	Not demonstrated as an integrated online DT	Explicit material wear	Wear projection	Parameter dependence and computational cost
Thermal instability models [45,46]	Thermo-mechanical analysis	Mainly offline	Hot spots and buckling conditions	Instability assessment	Internal-state observability and computational demand
Clutch degradation and diagnosis [10,120]	Stochastic degradation and vibration features	Method-dependent	Degradation indicators or fault classes	RUL in [10]; diagnosis in [120]	Limited continuous damage-state quantification
Transmission-related DT [152,153]	Dynamic and wear models with measurement feedback	Demonstrated in related systems	Gear fatigue and wear states	Damage and wear prediction	Transferability to wet clutches unverified
Stochastic PHM and decision models [161,162,164,166]	Degradation processes and MDPs	Recursive updating possible	Model-dependent latent states	RUL and maintenance policies	Dependence on observations and decision assumptions
Conceptual SEDT (this review)	Integrated damage-aware DT framework	Proposed, not validated	Intended explicit damage states	Proposed diagnosis and prognosis	Integrated implementation remains unvalidated

**Table 10:** Illustrative wear-state prediction and sequential parameter updating using synthetic variable-load data.

Block	Pressure (MPa)	Sliding Speed (m/s)	Predicted Cumulative Wear (μm)	Synthetic Observation (μm)	Updated $\hat{\kappa }$ (×10^−4^)
1	1.0	7	3.50	4	1.1429
2	1.5	10	12.57	13	1.1818
3	2.0	13	28.36	29	1.2083

Note: Each block contains 10,000 engagements with an assumed sliding duration of 0.5 s per engagement. Predicted values represent cumulative wear before the corresponding observation-based update. All operating parameters and wear observations are synthetic and are not derived from experimental measurements.

## Data Availability

No new experimental datasets were generated or analyzed in this review. The synthetic inputs, assumptions, and numerical results of the illustrative calculation are fully reported in Section 5.5. No additional datasets are available for sharing.

## Nomenclature

The following terms are defined for clarity:

Damage state variable	A measurable or model-estimated quantity representing a specific irreversible material or structural change, such as wear depth, permanent deformation, or crack length.
Damage-aware sensing	The acquisition and interpretation of damage-sensitive measurements to support the estimation of underlying physical damage states.
Self-evolving digital twin (SEDT)	A conceptual DT capable of updating not only operating-state estimates and model parameters but also validated damage-model representations or knowledge-based diagnostic and decision rules as new evidence becomes available.

## Abbreviations

The following abbreviations are used in this manuscript:

DT	Digital twin
SEDT	Self-evolving digital twin
IoT	Internet of Things
CFM	Copper-based friction material
PFM	Paper-based friction material
COF	Coefficient of friction
SVMR	Support vector machine regression
HPC	Heat partition coefficient
TEI	Thermoelastic instability
PLM	Product lifecycle management
MSM	Mirrored spaces model
DTW	Digital twin workshop
EDT	Evolutionary digital twin
PHM	Prognostics and health management
MDP	Markov decision process
